# Transforming multimorbidity care: Organizational barriers and provider behaviour in type 2 diabetes and cardiovascular disease

**DOI:** 10.1111/bjhp.70091

**Published:** 2026-07-10

**Authors:** Jessica Emily Brown, Frederick Kanayo Umeh, Robyn Lotto, Reda Madroumi, Ian Jones, Amineh Rashidi, Lisa Newson

**Affiliations:** ^1^ School of Psychology Liverpool John Moores University Liverpool UK; ^2^ School of Nursing and Advanced Practice Liverpool John Moores University Liverpool UK; ^3^ Liverpool Centre for Cardiovascular Science Liverpool UK; ^4^ School of Nursing, Faculty of Health Sciences Curtin University Bentley Western Australia Australia

**Keywords:** Cardiovascular Diseases, Diabetes Mellitus, Type 2, Health Personnel, Integrated Care, multimorbidity, Qualitative Research

## Abstract

**Background:**

Healthcare professionals' behaviours are central to effective multimorbidity management yet remain underexplored in behavioural medicine. Co‐existing type 2 diabetes (T2D) and cardiovascular disease (CVD) present intertwined behavioural and biomedical challenges; however, the organizational and professional factors that shape integrated care are poorly understood.

**Objectives:**

The objective of this study was to identify behavioural and organizational determinants of integrated T2D–CVD care and to apply behaviour change theory to provider practice.

**Design:**

Sixteen healthcare professionals in North‐West England participated in semi‐structured interviews.

**Methods:**

Data were analysed inductively using reflexive thematic analysis within a critical realist framework. The COM‐B model (capability, opportunity, motivation, behaviour) informed interpretation of these inductive findings.

**Results:**

Three interconnected themes: Compartmentalized conditions; inhibition of meaningful interactions; and gap between understanding and supporting illustrate how limitations in capability (confidence and training), opportunity (siloed records, absence of psychological pathways) and motivation (risk aversion and entrenched norms) collectively reinforce fragmented biomedical care. These mechanisms operate across organizational and cultural boundaries and explain persistent gaps in risk communication, cross‐disciplinary collaboration and limited psychological support.

**Conclusions:**

This study provides a theory‐informed qualitative application of the COM‐B model to healthcare professional behaviour in multimorbidity care, demonstrating how system design and professional culture shape interacting determinants. Conceptualizing cardiometabolic care as a behavioural and communicative system identifies priority intervention targets: staff training, service redesign, interoperable records and leadership development. These support practitioner well‐being, interdisciplinary collaboration and patient engagement. The findings reframe integrated T2D–CVD care as a multidirectional capability model, informing policy and practice.

**Statement of Contribution**

**What is already known on this subject?**

- Care for people living with T2D and CVD multimorbidity is often fragmented, with siloed pathways and inconsistent communication of cardiovascular risk.
- Organizational constraints such as short appointments, incompatible records and limited access to psychological support hinder integrated, person‐centred multimorbidity care.
- Multimorbidity research has tended to centre on patients' self‐management, leaving healthcare professionals' everyday behaviours and how these interact with service design comparatively under‐examined.

**What does this study add?**

- Positions healthcare professionals' own behaviour as an active determinant of T2D–CVD care, not simply the context in which care happens.
- Identifies a consistent gap between clinicians recognizing psychological distress and being able to respond to it, with limited pathways, training and time leaving this need unmet.
- Shows how communication caution, time and template pressures and absent psychological‐care pathways interlock into a self‐reinforcing cycle that sustains fragmented, single‐disease care.

## INTRODUCTION

Multimorbid type 2 diabetes (T2D) and cardiovascular disease (CVD) present intertwined behavioural and biomedical challenges that require integrated behavioural medicine approaches. Behavioural medicine examines the reciprocal influences of biological processes, health behaviours and social context and seeks to develop interventions that modify both patient and professional behaviours to improve health outcomes (Dekker et al., 2021). Managing T2D and CVD therefore requires attention not only to pharmacological treatment and risk factor control but also to the communication practices, motivational strategies and organizational behaviours of the healthcare professionals (HCP) (Paiva et al., 2019).

Multimorbidity is defined as the co‐existence of two or more chronic conditions (National Institute for Health and Care Excellence [NICE], 2023). In the United Kingdom, more than 65% of adults aged 65 years or greater live with multimorbidity, a figure projected to rise to 67.8% by 2035 (Kingston et al., 2018). Globally, the prevalence of diabetes continues to rise, with more than 1.3 billion people expected to live with the condition by 2050, of which the majority will live with type 2 diabetes (Ong et al., 2023). Adults with T2D have a 1.5‐ to 2‐fold higher risk of developing cardiovascular disease than those without diabetes (Pearson‐Stuttard et al., 2021; Rawshani et al., 2018), and similar patterns are reported internationally. These conditions share overlapping metabolic and vascular pathways and are increasingly conceptualized within a broader cardiometabolic or cardiorenal‐metabolic disease cluster (Marassi & Fadini, 2023; McGuire et al., 2021). Effective management therefore requires coordinated care that addresses both biomedical risk factors and the behavioural processes influencing treatment adherence, lifestyle change and self‐management (Olateju et al., 2021).

Despite robust epidemiological evidence linking T2D with elevated cardiovascular risk, how these risks are communicated and addressed within routine care remains less well understood (Chia et al., 2025). HCP behaviours, including risk‐communication practices, motivational approaches and inter‐professional collaboration, play a central role in shaping patient understanding, engagement and self‐management (Fisher et al., 2017; Hohberg et al., 2025).

However, international surveys suggest that many individuals with T2D remain unaware of their cardiovascular risk (Saeedi et al., 2020), and qualitative studies examining how clinicians communicate and manage cardiometabolic risk within everyday practice remain limited (Jutterström et al., 2024). Organizational factors further complicate care delivery. Short consultations, incompatible electronic health records and siloed service structures can constrain opportunities for integrated communication and collaborative working (Moffat & Mercer, 2015). As a result, care often remains oriented around single‐disease management despite the interconnected nature of cardiometabolic conditions.

Psychological comorbidity further complicates multimorbidity care. Depression and emotional distress are common among people with T2D and are associated with poorer self‐management and increased cardiovascular risk. A meta‐analysis of 17 studies involving more than one million participants found that depression significantly increases both fatal and non‐fatal CVD events in individuals with T2D (Inoue et al., 2020). However, evidence suggests that psychological needs are often insufficiently addressed within routine diabetes care (Stoop et al., 2019). Furthermore, communication challenges, limited consultation time, unclear professional responsibilities and organizational factors constrain HCP ability to address psychological concerns alongside biomedical management (Paiva et al., 2019). Understanding how HCP perceive and respond to these intersecting physical and psychological needs is therefore important for developing effective behavioural medicine interventions.

Policy responses increasingly acknowledge the need for integrated approaches to multimorbidity. In England, the 10‐Year Health Plan emphasizes prevention‐focused, community‐based services for long‐term conditions such as T2D and CVD (Department of Health and Social Care, 2025). Similarly, international initiatives, including the Netherlands' Care Group model and Canada's Coordination of Professional Care for the Elderly (COPA) programme, aim to improve organizational coordination across chronic disease services (Melchiorre et al., 2018; Wankah et al., 2018). Nevertheless, health systems continue to prioritize single‐disease pathways, constraining the delivery of integrated and patient‐centred care for people with multimorbidity (Mercer et al., 2012; Moffat & Mercer, 2015).

To better understand the behavioural and organizational mechanisms shaping multimorbidity care, this study draws on the Capability, Opportunity, Motivation, Behaviour (COM‐B) framework (Michie et al., 2011). COM‐B conceptualizes behaviour as arising from interactions between individuals' capability, opportunity and motivation and has been widely applied in behaviour change research and intervention design. Here, COM‐B is used not as an a priori coding framework but as a sensitizing and interpretive lens (Braun & Clarke, 2022). It has informed the framing of the interview prompts and, following inductive theme development, provided a behavioural vocabulary for interpreting how professional and organizational conditions shape the delivery of integrated care for people living with T2D–CVD multimorbidity.

### Objective

This study explored HCP perspectives on managing T2D and CVD multimorbidity in community settings in North‐West England. Guided by a behavioural medicine lens and informed by the COM‐B framework (Michie et al., 2011), the study focused on HCPs delivery of integrated cardiometabolic (T2D–CVD) care as the target behaviour. Specifically, it examined how HCPs communicate cardiovascular risk, collaborate across disciplines and services and recognize and respond to patients' psychological support needs. Furthermore, the study examined how professional behaviours and system structures shape these components and, by identifying barriers and opportunities for change, the study aims to inform future behavioural medicine interventions and service development for people living with cardiometabolic multimorbidity.

## METHODOLOGY

### Design

This exploratory qualitative study employed reflexive thematic analysis (RTA) (Braun & Clarke, 2022), a flexible yet rigorous approach for examining patterns of meaning in experiential data. Guided by critical realist ontology (Bhaskar, 1978) and contextualist epistemology (Madill et al., 2000), we recognized that HCP experiences are shaped by both material health system structures and social contexts. RTA was selected for its capacity to support interpretive, theoretically informed analysis while remaining grounded in participants' accounts.

### Participants and sampling

Ethical approval was obtained from the National Health Service (NHS) Health Research Authority and Research Ethics Committee (REC) (23/NI/0054). All HCP were employed within NHS services and were eligible if they (a) provided care to adults with T2D and chronic coronary syndrome (CCS), including coronary artery disease, coronary heart disease, ischaemic heart disease, stable angina or previous acute coronary syndrome and (b) worked in community or hospital settings.

Using purposive sampling (Campbell et al., 2020), participants were recruited to capture a range of professional perspectives involved in cardiometabolic care, including roles in risk communication, lifestyle counselling, medicines optimization and psychosocial support. Sixteen HCP were recruited between June 2023 and February 2024. Thirteen participants worked in community‐based services and three in hospital settings. Participants were identified through a partner community‐care organization via email invitations and service noticeboards, supplemented by promotion through cardiology and nursing professional networks.

The final sample comprised five family doctors, four diabetes‐related nurses, three health coaches, two cardiac‐related nurses, one clinical pharmacist and one community advanced nurse practitioner with experience ranging from 6 months to 28 years (mean 10.4 years) (see Table 1). Socioeconomic status (SES) of the practising area was estimated using Census 2021: Households by Deprivation Dimensions data (Office for National Statistics, 2022). Each practice postcode was mapped to its corresponding Middle Layer Super Output Area (MSOA), and the percentage of households deprived in at least one dimension (employment, education, health/disability or housing) was recorded. Reported SES values therefore represent the deprivation at area level (e.g. 37.1%–46.9%), rather than individual SES. Sample size was guided by information power (Malterud et al., 2016) rather than data saturation, which is conceptually inconsistent with reflexive thematic analysis (Braun & Clarke, 2022). The specificity of the aim, the information‐rich accounts of an experienced, multidisciplinary sample, and the analytic depth supported by team dialogue indicated that 16 interviews held sufficient information power to address the study's interpretive aims.

**TABLE 1:** Participant characteristics.

HCP	Role	Years of experience	Community vs. hospital setting	Gender	Ethnicity	SES of practising area
1	CVD health coach lead	0.5 years	Community	Female	White British	37.1%–46.9%
2	Health coach	2 years	Community	Male	White British	37.1%–46.9%
3	Family doctor	20 years	Community	Female	White British	37.1%–46.9%
4	Family doctor	5 years	Community	Male	White British	37.1%–46.9%
5	Diabetic nurse	16 years	Community	Female	White British	37.1%–46.9%
6	Diabetic nurse	12 years	Community	Female	White British	37.1%–46.9%
7	Lead community cardiac nurse	6 years	Community	Female	White British	37.1%–46.9%
8	Cardiovascular nurse specialist	10 years	Hospital Setting	Female	White British	49.3%–55.5%
9	Clinical pharmacist	10 years	Community	Male	White British	37.1%–46.9%
10	Diabetic inpatient nurse	1.5 years	Hospital Setting	Female	White British	37.1%–46.9%
11	Family doctor	28 years	Community	Male	White British	37.1%–46.9%
12	Family doctor	4 years	Community	Female	British South Asian	37.1%–46.9%
13	Family doctor	11 years	Community	Female	White British	37.1%–46.9%
14	Diabetes specialist dietitian	6 Years	Hospital setting	Female	White British	37.1%–46.9%
15	Community advanced nurse practitioner	4 years	Community	Female	White British	37.1%–46.9%
16	Health coach	3 years	Community	Male	White British	37.1%–46.9%

### Data generation

Semi‐structured interviews were conducted face to face by the first author (June 2023 to February 2024), with only the interviewer and participant present. After obtaining written informed consent, participants discussed (1) professional background and role; (2) barriers and facilitators to delivering T2D–CVD care; and (3) approaches to addressing psychological needs.

The interview guide included prompts sensitized to the COM‐B model (behaviour arises from the interaction of capability, opportunity and motivation) (Michie et al., 2011) to illuminate behavioural determinants of practice and risk communication (e.g. ‘What skills or resources would help you feel more confident discussing cardiac risk with patients?’) (See File S1). The framework informed later stage interpretation while preserving inductive theme development. Consistent with an iterative approach, later interviews were informed by developing analytic insights and included more focused, interpretative prompts to explore insights in greater depth. Interviews lasted a mean of 32 min, were audio‐recorded on encrypted devices, transcribed verbatim and anonymized. All participants consented, and no one declined to take part. The interviewer, previously employed by one partner organization, had no direct relationship with participants and discussed positionality during consent to promote openness.

### Data analysis

Data generation and analysis proceeded iteratively. Analysis began after the first 13 interviews, which informed subsequent recruitment to extend diversity and depth. Data were analysed using RTA (Braun & Clarke, 2019, 2022). The analysis was recursive and interpretive, involving active engagement with the data through repeated reading, reflexive memo‐writing and the generation of inductive semantic and latent codes.

Consistent with RTA, analysis was not conducted as a linear sequence of stages but evolved through ongoing engagement with the dataset. Initial coding and candidate themes developed from the first set of interviews were treated as provisional. The inclusion of additional interviews enabled these developing interpretations to be interrogated, extended and refined, supporting deeper conceptual development across the dataset. These interpretations were captured through reflexive memos and team analytic discussions, which in turn informed the prompts used in later interviews (see File S2).

All 16 interviews were coded using note‐mapping and Microsoft Word's comment function. Codes were collated into candidate themes and iteratively developed through reflexive discussion between the first and last authors, with further refinement through engagement with the multidisciplinary research team. Subthemes were used to capture nuanced facets of each theme. Themes were generated from patterns of shared meaning across the dataset, rather than derived from pre‐existing theoretical categories. The COM‐B framework (Michie et al., 2011) informed later stage interpretation but did not structure coding or theme development. To further contribute to the analytical interpretation, the themes were discussed with a Patient and Public Involvement and Engagement (PPIE) representative (an 82‐year‐old male with lived experience of T2D and CVD, including a triple heart bypass). This contribution was treated as a reflexive and interpretative engagement with the analysis, rather than a process of validation, and offered additional perspectives on how the analysis resonated with patient experience.

The study followed the Reflexive Thematic Analysis Reporting Guidelines (RTARG) (Braun & Clarke, 2024). Full analytical details and the coding examples are provided in File S2 (see also File S4).

### Research team and reflexivity

The multidisciplinary team comprised a trainee health psychologist (first author), a Professor of Health Psychology and Behavioural Medicine (Supervisor and Lead), a Senior Lecturer metabolic‐health researcher, two senior cardiovascular nurses, a research associate in health psychology and diabetes research and a Professor of Cardiovascular Nursing. Collectively, the team brought expertise in cardiac and diabetes care, qualitative methodology and behavioural medicine.

We acknowledge that our commitment to patient‐centred practice may orient us towards viewing multimorbidity as a psychosocial as well as biomedical challenge. Reflexive memo‐writing and team discussions were used throughout the analytical process to surface, interrogate and critically reflect on these assumptions (Berger, 2015).

### Data management

Participants could consent for anonymized transcripts to be deposited in a secure data archive for secondary analysis (Brown et al., 2025b). File S3 provides extended anonymized quotations and analytical interpretations supporting each theme.

### Findings

Three interconnected themes captured how HCP navigate the complex needs of people living with T2D and CVD: (1) Compartmentalized conditions—fragmented services and uneven communication of CVD risk; (2) inhibition of meaningful interactions—structural and cultural barriers that limit holistic encounters; and (3) gap between understanding and supporting—recognition of psychological distress without commensurate action.

#### Theme 1: Compartmentalized conditions

This theme captures how structural separation and professional behaviour combine to keep T2D and CVD care in distinct ‘boxes’. HCP described parallel clinical pathways, limited cross‐disciplinary dialogue and a reluctance to communicate shared risk. Two subthemes, Disparities in CVD Communication and Segregated Care, illustrate how this compartmentalization begins in consultation and extends through service design.

##### Disparities in CVD communication

Community practitioners frequently described hesitation when raising cardiac risk during diabetes reviews, worrying that an already complex diagnosis might overwhelm patients. As one family doctor (HCP 4) reflected, ‘I probably try to… but it depends where the conversation goes’, while another admitted feeling they were ‘bombarding them’ with information (HCP 3). Several participants also noted that this caution leaves many patients unaware of cardiovascular risk: ‘By the time they come to us they've seen lots of practitioners but still don't know the risks’, noted the dietitian (HCP 14). To avoid ‘frightening’ people, some clinicians defaulted to generic lifestyle messages rather than explicitly linking T2D and CVD. A diabetes nurse (HCP 5) explained, ‘It's more about general lifestyle, smoking, alcohol, so they can be a healthier version of themselves’. Hospital‐based staff were frustrated by these omissions, observing that patients often arrived with preventable complications because ‘diabetes education at the start isn't strong enough to prevent cardiac issues’ (Diabetic In‐patient Nurse, HCP 10). A minority of participants, generally those with additional training, reported adopting the recently recognized integrated cardiometabolic‐renal perspective: ‘There's been a change, people are starting to see it as one approach, but many still focus only on HbA1c’, said a clinical pharmacist (HCP 9). Their accounts illustrate how enhanced capability can disrupt entrenched habits but also how uneven such progress remains.

##### Segregated care

Beyond the consultation, service structures reinforced the divide. Practitioners depicted parallel diabetes and cardiac pathways with limited cross‐disciplinary understanding. ‘We're specialists in cardiovascular health, not diabetes… we just refer back to the family doctor’, admitted a community cardiac nurse (HCP 7). In hospital settings, ‘two separate teams’ worked side‐by‐side but rarely together, prompting calls for regular joint meetings (HCP 10). Some clinicians considered routine biomarker checks sufficient to manage the overlap, ‘we check blood pressure and lipids during diabetes checks, so we are staying on top of it’, said one family doctor (HCP 13), yet others doubted patients truly grasped their risk, remarking that ‘compared to cancer, cardiovascular disease? Nobody [understands it]’ (HCP 9). These accounts highlight how fragmented pathways and limited shared learning perpetuate single disease thinking even when monitoring is in place.

##### Interpretation and link forward

Theme 1 highlights how multimorbidity care is shaped not only by biomedical complexity but also professional practices and organizational structures. Clinicians recognized the interdependence of T2D and CVD, yet hesitancy around risk communication, limited opportunities for joint working and entrenched single‐disease routines sustained fragmented care. These patterns illustrate how professional behaviour and system design co‐produce everyday multimorbidity management. This structural and behavioural compartmentalization shapes patient encounters and links them into Theme 2.

#### Theme 2: Inhibition of meaningful interactions

This theme examines how structural and organizational barriers curtail the depth and quality of clinical encounters once patients are in the consulting room. Building on the previous theme's account of compartmentalized services, the analysis shows how time‐limited appointments, disease‐specific templates and incompatible electronic systems reduce opportunities for relational and integrated care.

##### Fragmented appointment structure

Across professional groups, time pressure dominated accounts of daily practice. One family doctor (HCP 3) captured the prevailing sentiment: ‘The big barrier to personalised care is time’. Consultations designed for a single condition left little room for holistic discussion, and electronic templates reinforced the problem. As another GP (HCP 11) explained, ‘Patients come for three or four different reviews. If there were comorbidity clinics where everything was covered together, it would make sense’. Technology intended to streamline care often added to the burden. A cardiovascular health coach (HCP 1) described how ‘you rely too much on human touch… it would help if EMIS (electronic health record) could pull through a template for multiple conditions’. Together, these accounts illustrate how service design narrows the space for dialogue, even when clinicians are motivated to provide person‐centred support.

##### Disjointed collaboration

Participants also highlighted gaps between professional groups that impeded coordinated care. One GP (HCP 11) likened the system to ‘supporting different parts of the elephant… everyone's working in their own silos with no consensus on direction’. Hospital clinicians criticized what they saw as lax community management and inconsistent targets: ‘Average blood sugars of 47 are documented as fine; it's not fine because they're going to have a heart attack in five years’, warned an in‐patient diabetes nurse (HCP 10). Community staff, for their part, pointed to incompatible systems and slow communication: ‘We work on electronic records, they mainly use paper; we liaise via letters and patients fall in the middle’, noted another GP (HCP 12). An advanced nurse practitioner (HCP 15) further suggested that these technological and communication gaps contribute to fragmented care, explaining that ‘patients don't come in because they don't want to see two different people, and you don't know if they've had everything done’, highlighting how poor coordination can discourage engagement and leave care incomplete. Other community practitioners echoed the need for change, suggesting more flexible, patient‐driven options: ‘Give patients a menu of options, a range of human beings they can choose from’ proposed the health coach (HCP 2).

##### Interpretation and transition

Here, opportunity is the dominant constraint. Short consultations, rigid templates and disjointed information systems reduce chances for integrated dialogue and collaborative working. Over time, these conditions erode professional motivation and embed habits of single‐disease care even among clinicians who value holistic practice. These everyday service conditions are not merely operational challenges but actively constrain how care is delivered. This provides a context for understanding Theme 3.

#### Theme 3: Gap between understanding and supporting

This theme explores the disconnect between recognizing psychological distress and providing meaningful support for people living with multimorbidity. Building on the earlier themes of compartmentalized care and constrained consultation time, the current theme shows how HCP acknowledge the emotional toll of multimorbidity yet rarely translate this awareness into action.

##### Recognition without response

Participants consistently described the heavy psychological burden carried by this patient group. A cardiovascular nurse (HCP 8) observed, ‘Depression is huge… something you think about all the time and that's very difficult’, while a family doctor (HCP 3) noted that ‘people give up; life shrinks, and they do less because they're not supported to do more’. Such comments demonstrate clear capability to perceive distress and to link emotional well‐being with physical outcomes.

##### Passing responsibility

Despite this recognition, most HCP deflected responsibility for providing psychological care. A diabetes nurse (HCP 6) admitted, ‘We don't really get involved, we give information sheets and refer to the family doctor’, and a cardiovascular nurse (HCP 8) echoed, ‘I just refer back to family‐doctor land’. Where referrals were made, services often failed to meet need. One clinical pharmacist (HCP 9) described how ‘Talking Therapies isn't built for chronic illness… waits are long, and after a few sessions patients are discharged’. Others such as the diabetes specialist dietitian (HCP 14) confirmed this gap: ‘The psychologist can only see people with insulin anxiety, we need someone who looks at depression linked to diabetes’. A health coach (HCP 16) further expressed this frustration, noting that ‘sometimes the answers are simple, but right now, the system makes them complicated’, reflecting a broader sense that relatively straightforward psychological support is rendered difficult to access due to fragmented pathways and limited service provision.

##### Interpretation and transition

Theme 3 highlights a persistent gap between recognizing psychological distress and responding to it within routine multimorbidity care. Although HCP demonstrated clear capability to identify emotional burden, a lack of specialist care pathways and training for managing co‐occurring conditions, compounded by clinical uncertainty around responsibility and concerns about raising issues without follow‐up constrained action. Furthermore, fear of exacerbating distress or ‘opening a can of worms’ reinforced avoidance. Together, these dynamics suggest that organizational conditions may marginalize psychological needs within routine care, despite their well‐established influence on self‐management and cardiovascular risk.

##### Integrated summary of findings

The three themes form an interlocking narrative illustrating how system design and HCP behaviour sustain siloed care. Communication gaps (Theme 1) limit patients' understanding of the T2D–CVD connection and reduce readiness for self‐management. Time pressure, rigid templates and fractured information systems (Theme 2) further restrict opportunities for meaningful dialogue and collaboration. The absence of clear psychological pathways (Theme 3) discourages clinicians from addressing distress, even when recognized. Although the themes were generated inductively, their interpretation can be viewed through the COM‐B lens retrospectively. Theme 1 foregrounds constrained psychological capability and cautious motivation around risk communication. Theme 2 explains limited physical and social opportunity for collaborative working. Theme 3 illustrates how recognized capability is not enacted where opportunity (clear pathways) and motivation (role certainty) are lacking. Together, they create a pattern that may reinforce fragmented care practices in which professionals and structures co‐produce disjointed biomedical encounters despite broad recognition of the need for integration.

## DISCUSSION

This study highlights an under‐examined determinant of multimorbidity care: the everyday behaviours of HCP. The findings show that communication practices, responses to psychological need and patterns of inter‐professional working actively shape how patients understand risk, engage with treatment and manage long‐term conditions. Across the three themes, care was not only structured by clinical complexity but also by how professionals navigated and enacted their roles within organizational constraints.

This study contributes to behavioural medicine by applying the COM‐B framework (Michie et al., 2011) to HCP behaviour within cardiometabolic multimorbidity care. Although COM‐B has been used to understand professional practices in areas such as implementation and practice change (e.g. Atkins et al., 2017; Dyson & Cowdell, 2021; Mullan et al., 2022), its application to the management of multiple long‐term conditions remains limited. Prior applications have predominantly focused on discrete behaviours, often treating them in isolation. In contrast, the present analysis conceptualizes HCP behaviour as dynamically shaped through interacting capability, opportunity and motivation across professional, organizational and relational contexts. Using this framework as an interpretive lens, the findings illustrate how these interacting determinants shape HCP behaviour in practice. Across participants’ accounts, predominantly biomedical patterns of care were sustained by limited psychological capability, constrained opportunity and norm‐shaped motivation, even where participants recognised the psychological and behavioural dimensions of multimorbidity. This analysis illustrates how behavioural determinants operate across multiple levels of the care system, translating service‐level limitations into observable patterns of care delivery.

Despite the interconnected nature of these conditions (McGuire et al., 2021), participants described clinical systems that continue to structure care around single‐disease models, limiting opportunities for holistic risk communication and psychosocial support. From a behavioural medicine perspective, these organizational constraints shape HCP capacity to address multimorbidity as an integrated behavioural and clinical challenge.

These findings align with wider evidence highlighting persistent challenges in integrated care, including fragmented collaboration, limited interoperability and difficulties embedding psychological support within routine services (Melchiorre et al., 2018; OECD, 2023; Wankah et al., 2018). The present study builds on this literature by identifying the behavioural mechanisms through which these challenges are enacted in everyday clinical practice, showing how organizational conditions translate into patterns of professional behaviour and, in turn, patient experience.

Although international guidance increasingly advocates integrated management of T2D and CVD (ElSayed et al., 2023; Marx et al., 2023), few studies have examined how clinicians' communication practices, cross‐service collaboration and responses to psychological need shape patients' understanding, self‐management and long‐term risk. Participants' accounts suggest that this integration is not consistently realized in practice. These findings extend existing literature by showing how HCP behaviour and system design are active drivers of care quality. This is particularly relevant in culturally diverse contexts, where differing expectations around diet, family roles and self‐management can further complicate care delivery (Patel, Umeh, Poole, Vaja, & Newson, 2021).

The analysis identifies a coherent behavioural pathway in which structural features of care, including fragmented pathways, time‐limited consultations and limited psychological support, constrain HCP opportunity and, over time, shape capability and motivation to deliver integrated, psychologically informed care. As a result, psychological needs may be recognized but are not consistently acted upon. Table 2 extends this interpretation by outlining how these mechanisms can be targeted through coordinated changes in capability, opportunity and motivation across workforce, patient and system levels.

**TABLE 2:** Behavioural mechanisms and anticipated system‐level impacts in multimorbidity care.

Priority/intervention	Behavioural mechanism (COM‐B)	Intermediate outcomes	Potential impacts (workforce, patient, system)
Building capability (training in risk communication, shared decision‐making, motivational interviewing and acceptance and commitment therapy)	May support psychological capability and confidence for complex risk and emotional conversations	• Greater consistency in communication • Improved recognition of psychological needs • Increased cultural sensitivity	• Stronger therapeutic alliance and patient engagement (Brown, Newson, et al., 2025a) • Reduced emotional strain and burnout • Increased professional satisfaction and competence (NHS England, 2019, 2023; Rashidi et al., 2021)
Increasing opportunity (multimorbidity clinics, interoperable electronic health records, joint appointments, embedded psychological support)	May create physical and social opportunity for structured, collaborative care	• Fewer duplicate appointments • Improved continuity and inter‐professional collaboration	• More equitable access (Lotto et al., 2022; Rashidi, Whitehead, Halton, et al., 2025) • Reduced workload pressures • Improved job satisfaction, retention, and adherence outcomes
Strengthening motivation and culture (leadership incentives, cross‐boundary reflection and peer‐learning structures)	May strengthen reflective motivation and shared ownership of psychosocial and biomedical goals	• Greater openness to innovation • Stronger team identity • Normalization of psychologically informed practice	• Improved morale and retention (Brown, Newson, et al., 2025a; NHS England, 2023) • Reduced presenteeism and absenteeism • Stronger communication and safety culture
Enhancing patient communication and cognitive capability (structured conversation guides, self‐monitoring tools and cognitive‐behavioural therapy‐based support)	May support development of psychological capability and reflective motivation for effective self‐management	• Clearer understanding of illness and priorities • Increased participation in shared decision‐making	• Reduced anxiety (Brown, Newson, et al., 2025a; Rashidi, Whitehead, Newson, & Begum, 2025) • Improved adherence and quality of life (Newson et al., 2023)

### Policy and practice implications

The findings suggest three interrelated priorities for improving T2D–CVD long‐term condition management: strengthening capability, increasing opportunity and supporting motivation and professional culture. These priorities are interdependent and reflect the behavioural conditions shaping care in practice.

These priorities can be situated within the Behaviour Change Wheel (BCW), in which COM‐B sits at the hub and behavioural determinants are translated into intervention functions (Michie et al., 2011, 2014). The capability, opportunity and motivation constraints identified here point towards intervention functions such as education and training (risk communication and psychologically informed skills), environmental restructuring and enablement (multimorbidity clinics, interoperable records, embedded psychological pathways) and modelling and persuasion to shift professional norms. Full specification of intervention functions, policy categories and behaviour change techniques is the focus of ongoing work by the research team and Table 2 summarizes the determinant‐to‐priority logic that such development would build upon.

Strengthening capability involves supporting HCP to communicate complex, emotionally salient risk information and respond to psychological need. This may include training in risk communication, shared decision‐making (Elwyn et al., 2012; Joseph‐Williams et al., 2014) and brief psychologically informed approaches such as motivational interviewing or acceptance and commitment therapy (Rashidi et al., 2021; Zhang et al., 2018). Such approaches may increase confidence and consistency in addressing both biomedical and psychosocial aspects of care. Increasing opportunity requires changes to service redesign and infrastructure. Participants' accounts suggest that time‐limited consultations, disease‐specific templates and fragmented information systems constrain integrated working. Approaches such as multimorbidity clinics, joint appointments, interoperable electronic records and embedded psychological support may enable more coordinated and continuous care while reducing duplication.

Supporting motivation and professional culture involves shifting norms and expectations that shape clinical practice. Participants described a cautious orientation towards raising psychological concerns, particularly where follow‐up pathways were unclear. Creating opportunities for cross‐disciplinary reflection, recognizing collaborative practice and supporting peer‐learning may help to normalize psychologically informed care and reduce the emotional burden associated with complex consultations (Brown, Newson, et al., 2025a; Rashidi, Whitehead, Halton, et al., 2025).

Importantly, improving multimorbidity care cannot rest solely on HCP. Evidence from our wider programme suggests that patients and caregivers also require support to develop these behavioural conditions for effective self‐management and engagement. People living with T2D and CVD may struggle to connect their conditions, navigate competing treatment demands or articulate their needs (Brown, Newson, et al., 2025a; Rashidi, Whitehead, Halton, et al., 2025; Rashidi, Whitehead, Newson, & Begum, 2025), while caregivers often experience uncertainty and emotional strain (Newson et al., 2023). Supporting patients through structured communication tools, self‐monitoring strategies and psychologically informed interventions may enable more productive interactions between patients and professionals. This reflects a broader ‘two‐way capability’ perspective, in which collaborative care delivery depends on both well‐supported staff and supported patients.

While these recommendations align with current policy directions, their implementation must be considered within the constraints of healthcare systems. Resource limitations, workforce pressures and organizational complexity may limit the feasibility of large‐scale service redesign. In this context, lower cost interventions, such as structured communication prompts, targeted training and incremental improvements to digital services, may offer more achievable progress towards coordinated care.

The findings also have implications for health equity. Cardiometabolic multimorbidity disproportionately affects socioeconomically deprived and ethnic minority populations, who experience substantially higher cardiovascular risk and may face barriers such as dietary conflicts and culturally embedded practices (NHS England, 2019, 2023; Patel, Umeh, Poole, Vaja, & Newson, 2021; Patel, Umeh, Poole, Vaja, Ramtoola, & Newson, 2021). Standardized approaches may not address these complexities, risking the reproduction of existing inequalities and highlighting the need for culturally responsive, tailored support. These priorities also align with key policy frameworks, including the NHS Long Term Plan (NHS England, 2019), the NHS Long Term Workforce Plan (NHS England, 2023) and international guidance (Marx et al., 2023; OECD, 2023; Vrints et al., 2024; WHO, 2021).

### Strengths and limitations

A key strength of this study lies in its explicit use of a behavioural framework to explore HCP roles in care for people with multimorbidity, integrating psychological theory with everyday organizational practice. We adopted RTA within a critical realist framework and followed Braun and Clarke's (2025) quality principles, which supported a transparent, reflexive and analytically rich process. The purposive sample of 16 professionals (including family doctors, specialist nurses, pharmacists, dietitians and health coaches) working across community and hospital settings provided a range of perspectives that strengthened the breadth and potential transferability of findings. The research team combined academic behavioural science and clinical expertise; this mix supported ongoing reflexive dialogue that helped us examine our interpretations closely and consider alternative readings of the data. Including such varied professional viewpoints also deepened our understanding of communication, coordination and psychological integration in complex chronic care. A further strength comes from a PPIE representative with lived experience of T2D and CVD, whose feedback offered resonance with patient realities and helped ensure the final themes felt meaningful in real‐world context (Brett et al., 2014).

Several limitations should be noted. Recruitment was confined to a single UK region, and despite efforts to include hospital clinicians, the final sample was weighted towards community practitioners. Experience in areas with different commissioning arrangements, better electronic record sharing or alternative payment models may therefore vary. Following Braun and Clarke's (2022) guidance on data adequacy rather than saturation, the depth of analysis and the range of professional roles represented provided strong conceptual insight, outweighing the limits of numerical generalizability. The first author's existing connection to the recruiting organization may have shaped who chose to participate and what they felt comfortable sharing; however, reflexive practice, full anonymization and independent review by the wider team helped mitigate this (Berger, 2015). Finally, interviews were necessarily brief due to clinical workloads; longer or repeated encounters might have provided greater insight into how behaviours and service structures evolve over time.

### Future research

Future research should advance behavioural integration of multimorbidity care through three complementary strands. First, this programme has already moved through intervention development and early testing. The team co‐created an intervention with people living with T2D–CVD multimorbidity through workshops structured around the COM‐B model and the Behaviour Change Wheel (Brown et al., 2026; Brown, Umeh, et al., 2025), and a small‐scale acceptability study has since been completed; the underpinning deductive analysis, drawing on both COM‐B and the Theoretical Domains Framework, is reported separately (in preparation). The priority now is feasibility and effectiveness testing, ahead of a full randomized controlled trial. Second, multimorbidity clinics and joined‐up care pathways should be evaluated using hybrid effectiveness implementation designs that incorporate patient‐reported outcomes (e.g. distress, self‐management capability) and RE‐AIM framework, whose attention to reach, adoption, implementation and maintenance is well suited to evaluating the real‐world scalability, cost‐effectiveness and equity of service‐level change (Glasgow et al., 1999, 2019). Third, cross‐national comparison (e.g. UK–Netherlands–Canada) is warranted because these systems differ markedly in how integration is structured and financed, for example, through the Netherland's bundled‐payment Care Groups and Canada's COPA coordination model (Melchiorre et al., 2018; Wankah et al., 2018). Such comparison would clarify how system architecture and incentives shape provider capability, opportunity and motivation. Collectively, this work will contribute to developing equitable, sustainable and psychologically informed care for complex patient needs.

## CONCLUSION

This study reconceptualizes emerging cardiometabolic models, viewing them not merely as pharmacological algorithms but as cross‐disciplinary behavioural and communicative systems. Effective multimorbidity care depends on clinicians who can communicate shared risk, collaborate across disease silos and integrate psychological and biomedical management within everyday practice. In examining these behavioural mechanisms, this study draws on COM‐B as an interpretive lens for making sense of provider behaviour in a multimorbidity context. Previous COM‐B applications in diabetes or cardiovascular care have largely addressed single‐condition self‐management or patient‐level behaviour change (Murphy et al., 2023; Whittal et al., 2021). The present study applies the framework to a multimorbidity perspective, to understand inter‐professional and system‐level behaviours across the interconnected cardiometabolic spectrum. In this context, capability, opportunity and motivation help to illuminate the organizational and communicative dependencies that underpin, or undermine, integrated care. Integrated care is, moreover, a two‐way capability model, requiring reciprocal investment in workforce training and patient empowerment to achieve genuinely person‐centred care. Embedding these mechanisms in service design is likely to strengthen workforce well‐being, reduce burnout and improve job satisfaction, key enablers of high‐quality, equitable and sustainable healthcare. The mechanisms identified here offer a potentially transferable behavioural framework for the design and evaluation of integrated care interventions across other multimorbidity clusters such as COPD‐CVD and cancer survivorship. By bridging behavioural theory with real‐world multimorbidity practice, this study provides a foundation for aligning health system reform, professional culture and patient engagement, with potential implications for health equity, workforce sustainability and scalable models of integrated care.

## TRANSPARENCY STATEMENT

Study registration: This study was preregistered and approved by the National Health Service (NHS) Health Research Authority (Reference: 23/NI/0054) and also by Liverpool John Moores University Research Ethics Committee, which maintained oversight throughout the project. The preregistered protocol detailed the study objectives, design, sampling strategy and analytic framework. Analytic Plan Registration: The preregistered protocol specified the use of reflexive thematic analysis (Braun & Clarke, 2022) within a critical realist framework. Analytic procedures were implemented as planned and reported in File S2, consistent with the Reflexive Thematic Analysis Reporting Guidelines (RTARG; Braun & Clarke, 2024).

## AUTHOR CONTRIBUTIONS

**Jessica Emily Brown:** Conceptualization; methodology; investigation; formal analysis; writing – original draft; writing – review and editing; project administration. **Frederick Kanayo Umeh:** Conceptualization; supervision; methodology; validation; writing – review and editing. **Robyn Lotto:** Conceptualization; methodology; supervision; writing – review and editing. **Reda Madroumi:** Writing – review and editing. **Ian Jones:** Conceptualization; methodology; supervision; writing – review and editing. **Amineh Rashidi:** Validation; writing – review and editing. **Lisa Newson:** Conceptualization; methodology; validation; supervision; formal analysis; project administration; funding acquisition; writing – original draft; writing – review and editing.

## FUNDING INFORMATION

This study was funded by Liverpool John Moores University.

## CONFLICT OF INTEREST STATEMENT

The authors declare no conflicts of interest.

## Supporting information


**File S1.** Semistructured interview schedule.
**File S2.** Reflexive thematic analysis—coding and theme development.
**File S3.** All participants' quotes by theme and subtheme.
**File S4.** Author evaluation tool using the reflexive thematic analysis reporting guidelines.

## Data Availability

Anonymized transcripts and supporting materials are archived with the UK Data Service (SN 857712; https://doi.org/10.5255/UKDA‐SN‐857712) under restricted access. Data are available to accredited researchers upon reasonable request and approval from the corresponding author. Code availability: No computational code was used. The complete coding framework and analytic process are provided in File S2. Materials availability: All study materials, including participant information sheets, consent forms and interview guides, are available from the open‐access repository (as provided with data) and please see Supporting Information.

## References

[bjhp70091-bib-0001] Atkins, L. , Francis, J. , Islam, R. , O'Connor, D. , Patey, A. , Ivers, N. , Foy, R. , Duncan, E. M. , Colquhoun, H. , Grimshaw, J. M. , Lawton, R. , & Michie, S. (2017). A guide to using the theoretical domains framework of behaviour change to investigate implementation problems. Implementation Science: IS, 12, 77. 10.1186/s13012-017-0605-9 28637486 PMC5480145

[bjhp70091-bib-0002] Berger, R. (2015). Now I see it, now I don't: Researcher's position and reflexivity in qualitative research. Qualitative Research, 15(2), 219–234. 10.1177/1468794112468475

[bjhp70091-bib-0003] Bhaskar, R. (1978). A realist theory of science (2nd ed.). Harvester Press.

[bjhp70091-bib-0004] Braun, V. , & Clarke, V. (2019). Reflecting on reflexive thematic analysis. Qualitative Research in Sport, Exercise and Health, 11(4), 589–597. 10.1080/2159676X.2019.1628806

[bjhp70091-bib-0005] Braun, V. , & Clarke, V. (2022). *Thematic analysis: A practical guide to understanding and doing it* (Chapter 5). In Thematic analysis: A practical guide (pp. 87–110). Sage Publications. 10.4135/9781529712978.n5

[bjhp70091-bib-0006] Braun, V. , & Clarke, V. (2024). Supporting best practice in reflexive thematic analysis reporting in Palliative Medicine: A review of published research and introduction to the Reflexive Thematic Analysis Reporting Guidelines (RTARG). Palliative medicine, 38(6), 608–616. 10.1177/02692163241234800 38469804 PMC11157981

[bjhp70091-bib-0007] Braun, V. , & Clarke, V. (2025). Reporting guidelines for qualitative research: A values‐based approach. Qualitative Research in Psychology, 22(2), 399–438. 10.1080/14780887.2024.2382244

[bjhp70091-bib-0008] Brett, J. , Staniszewska, S. , Mockford, C. , Herron‐Marx, S. , Hughes, J. , Tysall, C. , & Suleman, R. (2014). Mapping the impact of patient and public involvement on health and social care research: A systematic review. Health Expectations, 17(5), 637–650. 10.1111/j.1369-7625.2012.00795.x 22809132 PMC5060910

[bjhp70091-bib-0009] Brown, J. , Newson, L. , Umeh, K. , Lotto, R. , & Jones, I. (2025b). Supporting those with multimorbidity: A qualitative study on the perspectives of healthcare professionals who provide care to individuals with type 2 diabetes and cardiovascular disease, 2023–2024 . [Data set], UK Data Service. 10.5255/UKDA-SN-857712

[bjhp70091-bib-0010] Brown, J. E. , Newson, L. , Lotto, R. , Umeh, K. , & Jones, I. (2026). The co‐creation of an intervention to enhance quality of life and psychological outcomes for those who live with type 2 diabetes and cardiovascular disease as a multimorbidity . [Oral Presentation]. British Psychological Society Division of Health Psychology Annual Conference, Liverpool, UK.

[bjhp70091-bib-0011] Brown, J. E. , Newson, L. , Umeh, K. , Lotto, R. , & Jones, I. (2025a). Living with multimorbidity: A qualitative study on the personal perspectives of individuals with type 2 diabetes and cardiovascular disease. Psychology & Health, 1–27. 10.1080/08870446.2025.2546411 40827896

[bjhp70091-bib-0012] Brown, J. E. , Umeh, K. , Lotto, R. , Jones, I. , & Newson, L. (2025). The co‐creation of an intervention to enhance outcomes for those who live with type 2 diabetes and cardiovascular disease . [Oral Presentation]. International Congress of Behavioral Medicine, Vienna, Austria.

[bjhp70091-bib-0013] Campbell, S. , Greenwood, M. , Prior, S. , Shearer, T. , Walkem, K. , Young, S. , Bywaters, D. , & Walker, K. (2020). Purposive sampling: Complex or simple? Research case examples. Journal of Research in Nursing, 25(8), 652–661. 10.1177/1744987120927206 34394687 PMC7932468

[bjhp70091-bib-0014] Chia, A. W.‐Y. , Teo, W. L.‐L. , Acharyya, S. , Munro, Y. L. , & Dalan, R. (2025). Patient‐physician communication of health and risk information in the management of cardiovascular diseases and diabetes: A systematic scoping review. BMC Medicine, 23, 96. 10.1186/s12916-025-03873-x 39984943 PMC11846366

[bjhp70091-bib-0015] Dekker, J. , Amitami, M. , Berman, A. H. , Brown, H. , Cleal, B. , Figueiras, M. J. , Finney Rutten, L. J. , Fors, E. A. , Griva, K. , Gu, J. , Keyworth, C. , Kleinstäuber, M. , Lahmann, C. , Lau, J. T. F. , Leplow, B. , Li, L. , Malmberg Gavelin, H. , Mewes, R. , Mo, P. K. H. , … Nater, U. M. (2021). Definition and characteristics of behavioral medicine, and main tasks and goals of the international society of behavioral medicine—An international Delphi study. International Journal of Behavioral Medicine, 28(3), 268–276. 10.1007/s12529-020-09928-y 32909153 PMC8121730

[bjhp70091-bib-0016] Department of Health and Social Care . (2025). 10 year health plan for England: Fit for the future. Prime Ministers Office. ISBN 978–1–5286‐5807‐2. https://www.gov.uk/government/publications/10‐year‐health‐plan‐for‐england‐fit‐for‐the‐future

[bjhp70091-bib-0017] Dyson, J. , & Cowdell, F. (2021). How is the theoretical domains framework applied in designing interventions to support healthcare practitioner behaviour change? A systematic review. International Journal for Quality in Health Care, 33(3), mzab106. 10.1093/intqhc/mzab106 34279637

[bjhp70091-bib-0018] ElSayed, N. A. , Aleppo, G. , Aroda, V. R. , Bannuru, R. R. , Brown, F. M. , Bruemmer, D. , Collins, B. S. , Cusi, K. , Das, S. R. , Gibbons, C. H. , Giurini, J. M. , Hilliard, M. E. , Isaacs, D. , Johnson, E. L. , Kahan, S. , Khunti, K. , Kosiborod, M. , Leon, J. , Lyons, S. K. , … Gabbay, R. A. (2023). Introduction and methodology: Standards of care in diabetes. (2023). Diabetes Care, 46(Suppl 1), S1. 10.2337/dc23-Sint 36507647 PMC9810461

[bjhp70091-bib-0019] Elwyn, G. , Frosch, D. , Thomson, R. , Joseph‐Williams, N. , Lloyd, A. , Kinnersley, P. , Cording, E. , Tomson, D. , Dodd, C. , Rollnick, S. , Edwards, A. , & Barry, M. (2012). Shared decision making: A model for clinical practice. Journal of General Internal Medicine, 27(10), 1361–1367. 10.1007/s11606-012-2077-6 22618581 PMC3445676

[bjhp70091-bib-0020] Fisher, L. , Polonsky, W. H. , Hessler, D. , & Potter, M. B. (2017). A practical framework for encouraging and supporting positive behaviour change in diabetes. Diabetic Medicine: A Journal of the British Diabetic Association, 34(12), 1658–1666. 10.1111/dme.13414 28636745 PMC5687986

[bjhp70091-bib-0021] GBD 2021 Diabetes Collaborators . (2023). Global, regional, and national burden of diabetes from 1990 to 2021, with projections of prevalence to 2050: A systematic analysis for the global burden of disease study 2021. Lancet, 402(10397), 203–234. 10.1016/S0140-6736(23)01301-6 37356446 PMC10364581

[bjhp70091-bib-0022] Glasgow, R. E. , Harden, S. M. , Gaglio, B. , Rabin, B. , Smith, M. L. , Porter, G. C. , Ory, M. G. , & Estabrooks, P. A. (2019). RE‐AIM planning and evaluation framework: Adapting to new science and practice with a 20‐year review. Frontiers in Public Health, 7, 64. 10.3389/fpubh.2019.00064 30984733 PMC6450067

[bjhp70091-bib-0023] Glasgow, R. E. , Vogt, T. M. , & Boles, S. M. (1999). Evaluating the public health impact of health promotion interventions: The RE‐AIM framework. American Journal of Public Health, 89(9), 1322–1327. 10.2105/AJPH.89.9.1322 10474547 PMC1508772

[bjhp70091-bib-0024] Hohberg, V. , Lichtenstein, E. , Kreppke, J. N. , Zanitti, C. , Streckmann, F. , Gerber, M. , & Faude, O. (2025). Effects of lifestyle interventions to promote physical activity on physical activity and glycated hemoglobin in patients with type 2 diabetes: A systematic review and meta‐analysis: V. Hohberg et al. Sports Medicine, 55(5), 1165–1181. 10.1007/s40279-025-02184-8 40080359 PMC12106156

[bjhp70091-bib-0025] Inoue, K. , Beekley, J. , Goto, A. , Jeon, C. Y. , & Ritz, B. R. (2020). Depression and cardiovascular disease events among patients with type 2 diabetes: A systematic review and meta‐analysis with bias analysis. Journal of Diabetes and its Complications, 34(12), 107710. 10.1016/j.jdiacomp.2020.107710 32921574 PMC7467011

[bjhp70091-bib-0026] Joseph‐Williams, N. , Edwards, A. , & Elwyn, G. (2014). Power imbalance prevents shared decision making. BMJ, 348, g3178. 10.1136/bmj.g3178 25134115

[bjhp70091-bib-0027] Jutterström, L. , Stenlund, A. L. , Otten, J. , Lilja, M. , & Hellström Ängerud, K. (2024). Awareness of cardiovascular risk among persons with type 2 diabetes: A qualitative study. International Journal of Qualitative Studies on Health and Well‐Being, 19(1), 2294512. 10.1080/17482631.2023.2294512 38112175 PMC11737827

[bjhp70091-bib-0028] Kingston, A. , Robinson, L. , Booth, H. , Knapp, M. , & Jagger, C. (2018). Projections of multi‐morbidity in the older population in England to 2035: Estimates from the population ageing and care simulation (PACSim) model. Age and Ageing, 47(3), 374–380. 10.1093/ageing/afx201 29370339 PMC5920286

[bjhp70091-bib-0029] Lotto, R. R. , Newson, L. M. , Patel, T. , McGinn, E. , & Jones, I. D. (2022). Exploring the experiences of the cardiac rehabilitation journey among south Asian patients. British Journal of Cardiac Nursing, 17(1), 1–12. 10.12968/bjca.2021.0109 38812658

[bjhp70091-bib-0030] Madill, A. , Jordan, A. , & Shirley, C. (2000). Objectivity and reliability in qualitative analysis: Realist, contextualist and radical constructionist epistemologies. British Journal of Psychology, 91(1), 1–20. 10.1348/000712600161646 10717768

[bjhp70091-bib-0031] Malterud, K. , Siersma, V. D. , & Guassora, A. D. (2016). Sample size in qualitative interview studies: Guided by information power. Qualitative Health Research, 26(13), 1753–1760. 10.1177/1049732315617444 26613970

[bjhp70091-bib-0032] Marassi, M. , & Fadini, G. P. (2023). The cardio‐renal‐metabolic connection: A review of the evidence. Cardiovascular Diabetology, 22(1), 195. 10.1186/s12933-023-01937-x 37525273 PMC10391899

[bjhp70091-bib-0033] Marx, N. , Federici, M. , Schütt, K. , Müller‐Wieland, D. , Ajjan, R. A. , Antunes, M. J. , Christodorescu, R. M. , Crawford, C. , Di Angelantonio, E. , Eliasson, B. , Espinola‐Klein, C. , Fauchier, L. , Halle, M. , Herrington, W. G. , Kautzky‐Willer, A. , Lambrinou, E. , Lesiak, M. , Lettino, M. , DK, M. G. , … ESC Scientific Document Group . (2023). 2023 ESC guidelines for the management of cardiovascular disease in patients with diabetes: Developed by the task force on the management of cardiovascular disease in patients with diabetes of the European Society of Cardiology (ESC). European Heart Journal, 44(39), 4043–4140. 10.1093/eurheartj/ehad192 37622663

[bjhp70091-bib-0034] McGuire, D. K. , Shih, W. J. , Cosentino, F. , Charbonnel, B. , Cherney, D. Z. I. , Dagogo‐Jack, S. , Pratley, R. , Greenberg, M. , Wang, S. , Huyck, S. , Gantz, I. , Terra, S. G. , Masiukiewicz, U. , & Cannon, C. P. (2021). Association of SGLT2 inhibitors with cardiovascular and kidney outcomes in patients with type 2 diabetes: A meta‐analysis. JAMA Cardiology, 6(2), 148–158. 10.1001/jamacardio.2020.4511 33031522 PMC7542529

[bjhp70091-bib-0035] Melchiorre, M. G. , Papa, R. , Rijken, M. , van Ginneken, E. , Hujala, A. , & Barbabella, F. (2018). eHealth in integrated care programs for people with multimorbidity in Europe: Insights from the ICARE4EU project. Health Policy, 122(1), 53–63. 10.1016/j.healthpol.2017.08.006 28899575

[bjhp70091-bib-0036] Mercer, S. W. , Gunn, J. , Bower, P. , Wyke, S. , & Guthrie, B. (2012). Managing patients with mental and physical multimorbidity. BMJ, 345, e5559. 10.1136/bmj.e5559 22945951

[bjhp70091-bib-0037] Michie, S. , Atkins, L. , & West, R. (2014). The behaviour change wheel: A guide to designing interventions. Silverback Publishing. ISBN: 978–1–912141‐00‐5.

[bjhp70091-bib-0038] Michie, S. , van Stralen, M. M. , & West, R. (2011). The behaviour change wheel: A new method for characterising and designing behaviour change interventions. Implementation Science, 6, 42. 10.1186/1748-5908-6-42 21513547 PMC3096582

[bjhp70091-bib-0039] Moffat, K. , & Mercer, S. W. (2015). Challenges of managing people with multimorbidity in today's healthcare systems. BMC Family Practice, 16, 129. 10.1186/s12875-015-0344-4 26462820 PMC4604728

[bjhp70091-bib-0040] Mullan, B. , Liddelow, C. , Haywood, D. , & Breare, H. (2022). Behavior change training for health professionals: Evaluation of a 2‐hour workshop. JMIR Formative Research, 6(11), e42010. 10.2196/42010 36399382 PMC9719063

[bjhp70091-bib-0041] Murphy, K. , Berk, J. , Muhwava‐Mbabala, L. , McHugh, S. , & Kearney, P. M. (2023). Using the COM‐B model and behaviour change wheel to develop a theory and evidence‐based intervention for women with gestational diabetes (IINDIAGO). BMC Public Health, 23, 894. 10.1186/s12889-023-15586-y 37189143 PMC10186807

[bjhp70091-bib-0042] National Institute for Health and Care Excellence . (2023). Multimorbidity . Clinical knowledge summary. Retrieved October 22, 2025, from https://cks.nice.org.uk/topics/multimorbidity/

[bjhp70091-bib-0043] Newson, L. , Brown, J. E. , & Dugdale, S. (2023). Being the supporter: An interpretative phenomenological analysis of the role of caregivers in the self‐management of type 2 diabetes. Psychology & Health, 40(3), 377–393. 10.1080/08870446.2023.2231004 37394809

[bjhp70091-bib-0044] NHS England . (2019). The NHS long term plan. https://www.longtermplan.nhs.uk/

[bjhp70091-bib-0045] NHS England . (2023). NHS long term workforce plan . https://www.england.nhs.uk/long‐read/nhs‐long‐term‐workforce‐plan‐2/

[bjhp70091-bib-0046] Office for National Statistics . (2022). Households by deprivation dimensions . (Dataset TS011, edition 2021, version 6) [Data set]. https://www.ons.gov.uk/datasets/TS011/editions/2021/versions/6

[bjhp70091-bib-0047] Olateju, I. V. , Ogwu, D. , Owolabi, M. O. , Azode, U. , Osula, F. , Okeke, R. , & Akabalu, I. (2021). Role of behavioral interventions in the management of obesity. Cureus, 13(9), e18080. 10.7759/cureus.18080 34671541 PMC8522530

[bjhp70091-bib-0048] Organisation for Economic Co‐operation and Development (OECD) . (2023). Health at a glance 2023: OECD indicators. OECD Publishing. 10.1787/7a7afb35-en

[bjhp70091-bib-0049] Paiva, D. , Abreu, L. , Azevedo, A. , & Silva, S. (2019). Patient‐centered communication in type 2 diabetes: The facilitating and constraining factors in clinical encounters. Health Services Research, 54(3), 623–635. 10.1111/1475-6773.13126 30815858 PMC6505418

[bjhp70091-bib-0050] Patel, T. , Umeh, K. , Poole, H. , Vaja, I. , & Newson, L. (2021). Cultural identity conflict informs engagement with self‐management behaviours for south Asian patients living with type‐2 diabetes: A critical interpretative synthesis of qualitative research studies. International Journal of Environmental Research and Public Health, 18(5), 2641. 10.3390/ijerph18052641 33807965 PMC7967381

[bjhp70091-bib-0051] Patel, T. , Umeh, K. , Poole, H. , Vaja, I. , Ramtoola, S. , & Newson, L. (2021). Health professionals' interface with cultural conflict in the delivery of type 2 diabetes care. Psychology & Health, 36(2), 230–248. 10.1080/08870446.2021.1960346 34351821

[bjhp70091-bib-0052] Pearson‐Stuttard, J. , Buckley, J. , Cicek, M. , & Gregg, E. W. (2021). The changing nature of mortality and morbidity in patients with diabetes. Endocrinology and Metabolism Clinics of North America, 50(3), 357–368. 10.1016/j.ecl.2021.05.001 34399950

[bjhp70091-bib-0053] Rashidi, A. , Whitehead, L. , Halton, H. , Newson, L. , Begum, S. , & Presseau, J. (2025). The changes in health‐related quality of life after attending cardiac rehabilitation: A qualitative systematic review of the perspective of patients living with heart disease. PLoS One, 20(1), e0313612. 10.1371/journal.pone.0313612 39883647 PMC11781667

[bjhp70091-bib-0054] Rashidi, A. , Whitehead, L. , Newson, L. , & Begum, S. (2025). A qualitative study of treatment adherence from the perspective of nurses and patients following acute coronary syndrome. BMC Nursing, 24(1), 1203. 10.1186/s12912-025-03469-z 41013469 PMC12465365

[bjhp70091-bib-0055] Rashidi, A. , Whitehead, L. , Newson, L. , Presseau, J. , & Begum, S. (2021). The role of acceptance and commitment therapy in cardiovascular and diabetes healthcare: A scoping review. International Journal of Environmental Research and Public Health, 18(15), 8126. 10.3390/ijerph18158126 34360420 PMC8345942

[bjhp70091-bib-0056] Rawshani, A. , Rawshani, A. , Franzén, S. , Sattar, N. , Eliasson, B. , Svensson, A. M. , Zethelius, B. , Miftaraj, M. , DK, M. G. , Rosengren, A. , & Gudbjörnsdottir, S. (2018). Risk factors, mortality, and cardiovascular outcomes in patients with type 2 diabetes. New England Journal of Medicine, 379(7), 633–644. 10.1056/NEJMoa1800256 30110583

[bjhp70091-bib-0057] Saeedi, P. , Petersohn, I. , Salpea, P. , Malanda, B. , Karuranga, S. , Unwin, N. , Colagiuri, S. , Guariguata, L. , Motala, A. A. , Ogurtsova, K. , Shaw, J. E. , Bright, D. , Williams, R. , & IDF Diabetes Atlas Committee . (2020). Global and regional diabetes prevalence estimates for 2019 and projections for 2030 and 2045: Results from the international diabetes federation diabetes atlas, 9th edition. Diabetes Research and Clinical Practice, 157, 107843. 10.1016/j.diabres.2019.107843 31518657

[bjhp70091-bib-0058] Stoop, C. , Pouwer, F. , Pop, V. , Den Oudsten, B. , & Nefs, G. (2019). Psychosocial health care needs of people with type 2 diabetes in primary care: Views of patients and health care providers. Journal of Advanced Nursing, 75(8), 1702–1712. 10.1111/jan.13996 30883846 PMC6850404

[bjhp70091-bib-0059] Vrints, C. , Andreotti, F. , Koskinas, K. C. , Aboyans, V. , Agewall, S. , Arbel, Y. , Barbato, E. , Bax, J. J. , Beer, B. N. , Bueno, H. , Cifkova, R. , Collet, J.‐P. , De Carlo, M. , Delgado, V. , Fitzsimons, D. , Gaemperli, O. , Gielen, S. , Hindricks, G. , James, S. , … Zeymer, U. (2024). 2024 ESC guidelines for the management of chronic coronary syndromes. European Heart Journal, 45(38), 3465–3578. 10.1093/eurheartj/ehae177 39210710

[bjhp70091-bib-0060] Wankah, P. , Couturier, Y. , Belzile, L. , Gagnon, D. , & Breton, M. (2018). Providers' perspectives on the implementation of mandated local health networks for older people in Québec. International Journal of Integrated Care, 18(2), 1–18. 10.5334/ijic.3098 PMC6095055 30127686

[bjhp70091-bib-0061] Whittal, A. , Störk, S. , Riegel, B. , & Herber, O. R. (2021). Applying the COM‐B behaviour model to overcome barriers to heart failure self‐care: A practical application of a conceptual framework for the development of complex interventions (ACHIEVE study). European Journal of Cardiovascular Nursing, 20(3), 261–267. 10.1177/1474515120957292 33909892

[bjhp70091-bib-0062] World Health Organization . (2021). WHO guideline on health workforce development, attraction, recruitment and retention in rural and remote areas . https://www.who.int/publications/i/item/9789240024229 34057827

[bjhp70091-bib-0063] Zhang, C. Q. , Leeming, E. , Smith, P. , Chung, P. K. , Hagger, M. S. , & Hayes, S. C. (2018). Acceptance and commitment therapy for health behavior change: A contextually driven approach. Frontiers in Psychology, 8, 2350. 10.3389/fpsyg.2017.02350 29375451 PMC5769281

