# Supplementary material for: Transforming multimorbidity care: Organizational barriers and provider behaviour in type 2 diabetes and cardiovascular disease
Source: Br J Health Psychol. 2026 Jul 10;31(3):e70091. doi: 10.1111/bjhp.70091 (PMC13354874; doi:10.1111/bjhp.70091)
Supplement: Supplementary file 1 — File S1. Semistructured interview schedule. File S2. Reflexive thematic analysis—coding and theme development. File S3. All participants' quotes by theme and subtheme. File S4. Author evaluation tool using the reflexive thematic analysis reporting guidelines. [file BJHP-31-0-s001.docx]

**Supplementary Files A - D**

File A: Semi-Structured Interview Schedule

File B: Reflexive Thematic Analysis - Coding and Theme Development

File C: All Participants Quotes by Theme and Subtheme

File D: Author Evaluation Tool Using the Reflexive Thematic Analysis Reporting Guidelines

**Supplementary File A: Semi-Structured Interview Schedule**

The interview guide was organized into three broad sections, with prompts sensitized to the COM-B model (Capability, Opportunity, Motivation-Behavior) to explore behavioral determinants of professional practice.

**Section 1 - Professional Background**

- Can you tell me a bit about yourself and your current job role?
- How long have you been working in this organization and in this role?
- What does a typical day look like for you in terms of patient care?

**Section 2 - Patient Care and Service Delivery**

- From your perspective, what are the key challenges in supporting people with type 2 diabetes and cardiovascular disease?
- Are there organizational or system-level factors that help or hinder integrated care?
- How do you communicate cardiovascular risk to patients with diabetes?
- What skills or resources would help you feel more confident discussing cardiac risk or psychological issues?

**Section 3 - Psychological Aspects of Care**

- What forms of psychological support are available for this patient group?
- How do you address mental health or emotional wellbeing in your consultations?
- Are there any barriers to providing psychological support within your service?

The schedule was used flexibly; follow-up questions were asked to explore issues raised by participants.

**Supplementary File B: Reflexive Thematic Analysis - Coding and Theme Development**

This file outlines the analytic process and provides an overview of coding and theme development.

**Phase 1: Familiarization**

- Repeated reading of transcripts and listening to audio recordings.
- Reflexive memos capturing initial impressions, emotional reactions, and potential codes.

**Phase 2: Coding**

- Line-by-line inductive coding developed manually, using paper-based notes and mind maps to visualize patterns and relationships.
- Codes were generated inductively from the data; COM-B was not applied at this stage. Its constructs informed interpretation only after inductive theme development was complete.

**Phase 3: Theme Construction**

- Codes collated into candidate themes reflecting patterns across the dataset.
- Initial themes reviewed and refined in team meetings to ensure conceptual clarity.

**Phase 4: Review and Refinement**

- Themes checked against full data set for coherence.
- Subthemes identified to capture nuanced aspects of each overarching theme.

**Reflexive engagement during data collection**

Data generation and analysis proceeded iteratively. After the first set of interviews, the first author maintained reflexive memos recording initial impressions, analytic hunches, and points of surprise or discomfort, alongside an audit trail of evolving interpretations. These memos were discussed with the supervisory lead and wider team in regular analytic meetings. Provisional insights arising from this process, for example, the recurring tension between recognising and acting on psychological distress, were used to refine and add more focused, interpretative prompts in later interviews, allowing developing interpretations to be interrogated and extended across the dataset. This reflexive engagement was treated as integral to analysis rather than as a separate verification step, consistent with the values of reflexive thematic analysis (Braun & Clarke, 2022).

In summary:

| **Theme development** | **Further team analytical discussions** | **Later phase participants** | **First author reflexive memos** | **Initial participants** |
| --- | --- | --- | --- | --- |
| **Compartmentalised Conditions** | Team discussions centred on how HCPs recognised the interrelationship between diabetes and CVD but often communicated and managed them separately. Debate focused on whether this reflects clinical pragmatism, role boundaries, or wider organisational structures. | Later participants reinforced concerns about fragmented care. One participant noted that patients attend separate disease clinics and therefore "don't make the connection between the two and how one can impact the other" (HCP13). Another described how clinicians continue "just focusing on the HbA1c rather than the bigger picture" (HCP9). | Reflections highlighted a contradiction whereby clinicians acknowledged cardiometabolic interdependence but often delivered care through disease-specific pathways. | HCPs consistently described a lack of communication of CVD risk: “we’re talking in a review for a chronic condition… more in general lifestyle” (HCP5) and acknowledged reluctance to explicitly link conditions due to concern about overwhelming patients. Reinforced by additional interview |
| **Inhibition of Meaningful Interactions** | Analytical discussions explored how organisational pressures shaped the content and quality of consultations. Time constraints and templates appeared to reduce opportunities for personalised discussions about risk and behaviour change. | Later participants continued to describe fragmented consultation structures. One argued that "the appointments are short, and everything is structured around ticking off biomedical boxes" (HCP 16). Another advocated multimorbidity clinics because current systems are organised around "disease profiles in a very separate way" (HCP13). | Memos focused on the tension between person-centred care ideals and target driven healthcare systems. | Time was consistently identified as a barrier: "the big barrier to all of this, and personalised care is time" (HCP3), while HCP5 stated, "it's never enough time, there's never enough." Participants also criticised templates as "a tick box exercise" (HCP3) that could prevent meaningful patient interaction (HCP5). |
| **Gap Between Understanding and Supporting** | Discussions explored how clinicians readily recognised emotional distress but often felt unable to address it within existing roles, skills, and resources. | Later participants reinforced this gap. One reported that clinicians "don't feel well equipped to address a patient's emotional needs and therefore feel reluctant to broach the topic" (HCP 14). Another reflected: "if someone is feeling hopeless or anxious, advice alone doesn't work" (HCP 15). | Memos suggested a recurring disconnect between recognising psychological burden and providing meaningful psychological support. | Participants frequently described anxiety, depression, guilt, and burnout. HCP 8 stated, "Diabetes depression is huge. Diabetes burnout is huge," while HCP5 described diabetes as "relentless" and associated with guilt around food choices. Despite recognising these impacts, HCPs often referred patients elsewhere for specialist support (HCP3, HCP6). |

**Final Themes**

1. **Compartmentalised Conditions**
   - Disparities in CVD Communication

Segregated Care

1. **Inhibition of Meaningful Interactions**
   - Fragmented Appointment Structure
   - Disjointed Collaboration
2. **Gap Between Understanding and Supporting**
   - Recognition Without Response
   - Passing Responsibility

**Supplementary File C: Extended Participant Quotations**

This file presents a broader set of anonymised quotations illustrating each theme.

**Theme 1: Compartmentalised Conditions**

- “Sometimes I feel like I’m working in a bubble, diabetes here, cardiology there, patients get mixed messages.” (Family Doctor)
- “I’m cautious because patients already have so much to manage; adding heart risk can feel overwhelming.” (Diabetes Nurse)

**Theme 2: Inhibition of Meaningful Interactions**

- “The electronic records don’t talk to each other; we waste time chasing letters.” (Community Nurse)
- “I call the hospital team directly if needed, it’s quicker than the formal route.” (Health Coach)

**Theme 3: Gap Between Understanding and Supporting**

- “Patients get anxious and depressed, but mental health services have long waits, so we just keep monitoring.” (Clinical Pharmacist)
- “Even brief motivational interviewing can help patients re-engage with their care.” (Health Coach)

**Supplementary File C: All Participants Quotes by Theme and Subtheme**

| Theme | Subtheme | Quote |
| --- | --- | --- |
| *Compartmentalised Conditions* | ***Disparities in CVD Communication*** | *Well everyone is different you know some could have more serious T2D and that takes us more down a serious route in terms of explaining risks but you know someone could just have made the numbers so perhaps we don’t explain the risks as much (HCP 12)*  *Erm we don’t want to scare them I suppose I think no consultation takes a set route and with this depends on where it goes (HCP 12)*  *Yes yes exactly and I really cant comment how they take the cardiovascular complication, I think because they have a lot to take in this doesn’t stay in their mind (HCP 12)*  *I don't think people are very aware about that and if they're diabetes is well controlled that that would mean that their cardiovascular system is at less risk (HCP 13)*  *They don’t realise that they've got something wrong and a lot of them have a little bit of understanding about diabetes, but don't realise how much impact it has on pretty much every organ in the body . So yeah, I mean, try to touch on things like the importance of you kind of lipid control, blood pressure control (HCP 3)*  *Ohh yeah, you like skin fall off, you're gonna go blind, you kidney's gonna fail. You have a heart attack………Its a lot (HCP 3)*  *Well, I mean, it's difficult because certainly with a diagnosis of diabetes, there's so much to think about. And I do feel that sorry for the patients, cause you've bombarding them with (information). (HCP 3)*  *Don't put it explicitly like that. I think I probably more say something like, you know, it's very important to look after your blood pressure and we need to, you know look after your your cholesterol as well. And now that you have type 2 diabetes because……..you're, you know, you're more at risk of a heart attack, and you may have a heart attack that doesn't cause pain and things like that…. I I think probably try to do that whether or not I do it very well. I'm not sure. It depends sometimes where the conversation goes (HCP 4)*  *Erm to be honest when you are discussing well I actually I would say yeah…..I think linking……perhaps…… linking heart disease with diabetes I don’t think *long pause* I don’t think I personally don’t think that I’m talking about heart disease when I talk about diabetes because I think there is enough in that appointment in what we discussed already is because diabetes is huge in itself but then you’re talking like kidney disease and then you have to say you could loose a toe….it’s a lot in general and then to talk about the heart so it’s like hang on so erm you’re talking about yeah so I would say I don’t go into heart I don’t mention the heart I don’t it would be absolutely too much (HCP 5)*  *Erm not in a way that its linked, but what we will be saying is we would not specially not in that way but what we also say that is that we’re talking about there in a errr errr there in a review for a chronic condition which a lot of the time is caused because of lifestyle in general so you’re so that’s that’s really what youre talking about in your diabetic review, are you smoking, how’s your alcohol, so its more in general lifestyle and lifestyle changes so they can be the healthier version of them (HCP 5)*  *I mean yeah yeah definitely, and this is why we try and make a goal that is achievable, and we tell the patient why it is we have to regulate the sugars and so we talk a bit about the eyes, heart and kidneys and then the mortality, but it does depend on the context of the consultation (HCP 12)*  *I think that is crucial if patients have that understanding or say your heart attack and your diabetes are actually linked. If we can control the one, it'll help the other. (HCP 9)*  *Yeah, I think I I feel that when you need to when you first when a diagnosis is first made because you know I can't talk about their medications without talking to to them about, you know, why you're taking the statins. Why is it important for you to take these tablets again? What is a long term, a long-term goal and part of our long-term goal is to reduce your cardiovascular disease risk and other complications. So you have I feel you have to have those conversations. (HCP 9)*  *So definitely I think from my perspective, it does feel like healthcare professionals are scared to actually communicate the risks as by the time they come to us they would of spoke to lots of different community practitioners but they still don’t know the risks associated (HCP 14)*  *Unfortunately, we see the worst cases where they end up, do having cardiac issues because they're diabetes, is poorly controlled and it's trying to tell them they don't have the education at the start of the diabetes journey to be able to prevent them from the cardiovascular issues (HCP 10)*  *No, but it but it has been a change, a lot of you know clinicians are set in their ways the sort of pre pre, pre the kind of new era that the cardiorenal approach that we're in over the last you know 5 years......So people do compare, compartmentalise it and you see that very often just focusing on the HbA1c rather than you know the bigger picture. So now I see it as one approach (HCP 9)*  *Yet I would say definitely and then when they do get cardiovascular issues, they have a lot of weight gain as well. And that usually causes the cardiovascular issues and then they end up having like angina, they get shortness of breath. Hmm yeah. Therefore, they're exercise is limited as well, and that's not helping the end up more weight and it just gets more difficult for them to manage their diabetes overall because now they're not thinking about just the diabetes, they're thinking about the cardiac side of it as well. So it's like sometimes in a catch 22, they're trying to do right for the diabetes, but the cardiac is preventing them as well (HCP 9)*  *Whether that's diabetes or cardiac or whatever. There needs to be. It needs to be. Better communication with the patient, what is what is our ultimate long-term goals here for you? But be specific around that. You know for, you know, if you have had a heart attack, we need to get your cholesterol at this level. All these medications need to be titrated up and the rationale behind it. Why are we doing this? Same with diabetes. You know, this is your sugar level. Now we want it here. Why do we want it like that? Because you know your micro and macro complications. Patients don't always understand the complications and the risks if they don't achieve these things. And some do and some don't care. But I mean, that is as long as patients make an informed decision about their care and their and their treatment. That's, you know, I feel that's your job (HCP 9)*  *So definitely I think from my perspective, it does feel like healthcare professionals are scared to actually communicate the actual risks as by the time they come to us they would of spoke to lots of different primary care practitioners but they still don’t know the risks associate. So in my job it's it's quite interesting because like I work in a multidisciplinary team. So by the time I see the patient, they would know why they're coming in, they would have seen a nurse, they would have seen a doctor, so they would have had (HCP 14)*  *Exactly. Yeah. No, there's no prevention. It's mostly just tackling worst cases. Like we see people when they're on incident. They've been into hospital and, you know, everything's happened to them (HCP 14)*  *I always link them together because they are linked together. High cholesterol levels are gonna cause higher blood sugar levels, but it's not it's not a given (HCP 14)* |
|  | ***Segregated Care*** | *We probably need to revisit that to do some teaching because we're specialist in cardiovascular health, but we're not specialist in diabetes, so sometimes we're only scratching the surface on it….and if we are concerned we just we just refer back to the family doctor because we're not management of diabetes we just make sure that their blood sugars are safe enough after heart failure for the medication usage. (HCP 7)*  *It's it's more kind of time all up together, assuming that they have both. Obviously, they don't have both. If we're looking at somebody just with cardiovascular disease and that are at higher chance of dying or something in the past of heart failure, it kind of previous strokes we would be looking again and their diabetes risk that would be part of the monitoring that we do with them anyway. (HCP 3)*  *Yeah. I mean, I think they probably don't so much. You know, they wouldn't come down probably sit there and say, you know, you know, I'm worried because I've got my diabetes that I'm gonna have a heart attack or stroke but It's probably not explicitly done separately. It's kind of holistically done as looking at all those factors and because they're such an overlap between the two conditions, it's quite easy to to look at both as part of a package rather than specifically say we'll have a look at your diabetes now and then now we're looking at your cardiovascular. (HCP 3)*  *Erm *long pause* look knowledge is power, you know so I think absolutely erm but I don’t know whether I wonder whether it’s a lot, there’s so much they are dealing with there kidneys, foot, eyes, and now you want to look at the heart and the thing is if we can change there lifestyle and improve there diabetes and hba1c then we improve there heart health anyway so instead adding more to the pot we need to, personally, we need to find a way and concentrate and find a way of making the diabetes alone a big enough incentive for them to change there lifestyle (HCP 5)*  *We'll see what medications they’re on but they are managed separately under the diabetes team in (insert location in northwest of England), which is a really, really good service. (HCP 7)*  *We're all involved in integrated care but I think we as community care answers for managing this and they'll do their recalls and follow ups then you've got your diabetes team then you've got your family doctor then they’re under another service so the patient gets fed up with it and they lose faith so the so community care yeah. (HCP 7)*  *Yeah I think linking the appointments would be good but I mean a lot of it is done as we check blood pressure, weight, but yeah a T2D review but highlighting more about the heart would be yeah would be a good idea (HCP 6)*  *I mean you’re looking at their cholesterol and blood pressure and hypertension link or CHD link we would update that, it’s all involved in the diabetes check anyway (HCP 6)*  *Yes I do erm but I’m not sure how it would work, people have lots of different comorbidities and it maybe too complex to create lots of them I’m not use, I mean I think diabetes and cardiovascular complications are looked at well as a comorbidity in care we check blood pressure, lipids at diabetes checks so we are keeping on top of it (HCP 12)*  *No I don’t feel it is segregated but erm erm so I think although I mean those with diabetes there main primary thing to look at is there diabetes but they also need to look at the cardiovascular affects, so checking their blood pressure, checking there lipids, and in that respect its separated but erm I think it’s more separated with other conditions, for example if you had asthma or lung disease they wouldn’t be looked at, at the same time and they would come back another time….but with diabetes the most important thing to look at would be there blood pressure even more so than there blood glucose so I feel those cardiometabolic things go together very naturally and erm it’s when other things such as osteoarthritis I feel that’s where there may be more disconnect (HCP 11)*  *Yeah well it forms part of core…erm routine care I suppose managing cardiovascular complications and risk factors so managing blood pressure is a core part it and looking at patient's kidneys and monitoring those and also looking after people's eyes and monitoring those and we get the regular eye screening checks (HCP 11)*  *Yes that should be part of usual care, and we check peoples feet and checking for any Peripheral vascular disease (HCP 11)*  *I mean I think diabetes is a build up to a lot of things relating to CVD such as hypertension you know liver function and it’s a lot of things in the background but yeah of course we see them together it’s very important to (HCP 12)*  *We probably need to revisit that to do some teaching because we're specialist in cardiovascular health, but we're not specialist in diabetes, so sometimes we're only scratching the surface on it….and if we are concerned we just we just refer back to family doctor because we're not management of diabetes we just make sure that their blood sugars are safe enough after heart failure for the medication usage (HCP 7)*  *we're not management of diabetes we we're just going to make sure that they are safe within when when looking at their heart failure but sometimes diabetes can make that hard because of the medications and if the patient is not compliant (HCP 7)*  *So people do compare, compartmentalise it and you see that very often just focusing on the hb1ac rather than you know the bigger picture. So now I see it as one one approach (HCP 9)*  *I would say they are definitely looked at from different angles, I mean it just goes to show, the incident rates for both. So for diabetes we have in house diabetes nurses, that are specialists in what they do, they have diabetic foot reviews, they have med reviews, reviews about their diabetes management in general, that you don’t tend to get that with CVD but I suppose it’s because it’s so big I want to say, you know, we do BP clinics and things like that and they are meant to have annual heart or if not ideally its meant to be every 6 months but they don’t have the capacity, and the annual BP reviews where you come in and have a conversation about how to manage your BP but that’s normally a nurse who would come in and take that appointment but I would say definitely the management of the two conditions sorry diseases there I would say 110% they are treated too separate (HCP 1)*  *I think that they all understand. I think people, the diabetes specialist nurses are really, really silo focused into diabetes. So they are getting better at kind of viewing it as a cardiovascular, metabolic renal, you know mash up multimorbidity’s area and so we think of like Sglt 2 GLP one receptor agonist and things that have good cardiovascular risk reduction as well as glycaemic lowering properties. They're really good at prescribing those in people now (HCP 8)*  *But as if you're looking at people holistically as well as in their other conditions, I think they're a bit afraid because they've got lack of experience in caring for people with cardiovascular disease. So adjusting like hypertension means they wouldn't. They probably wouldn't feel confident and competent in doing that. And that's fair enough, really. That isn't part of their current remit (HCP 8)*  *So when I went to diabetes and found a lot of it was very transferable, so a lot of complex Type 2 is people with cardiometabolic disease, which I was very used to seeing. So that was quite….. Felt quite natural. I thought I'd feel very unnatural to go into a different disease kind of area and it actually felt really I was OK once I got my head around the kind of medications and things for diabetes. I think it was quite an asset actually to have cardiovascular knowledge because they can treat the patient more holistically (HCP 8)*  *You know that is crucial, but patients are I don't think patients are educated enough to see that holistic approach and the same with with lipid control. Oh, nobody wants to have a stroke, but nobody wants to take a statin either. You know, again, broad generalisation, but it's like we don't do the holistic approach for patient (HCP 9)*  *they don’t really understand off the direct back the importance of disease management alongside purely the lifestyle take on it…a lot at that stage a lot feel they have to put reliance on medication for disease management. A lot of them don’t really understand how managing other comorbidities……like I don’t know erm ….maybe diabetes or I don’t know a multicohort of different comorbidities can directly impact your risk of developing further disease but also current disease management. (HCP 1)*  *But I don't think patients generally understand the risks of cardiovascular disease, of diabetes, you know, amputations that, you know, long term complications. Patients do not understand the risks. You know when you speak to anybody about cancer treatment, it's it's a very emotive subject, rightly. So don't get me wrong. But I think. Compared to cancer, cardiovascular disease? Nobody.” (HCP 9)*  *But I think it's it's not, it's not out there enough. I mean you know there's lots of, yeah, I mean, you know, people say well, the hypertension is one of the biggest killers. Yet people don't understand what that means because it's a gateway condition to so many other problems. (HCP 9)*  *I mean I think diabetes and cardiovascular complications are looked at well as a comorbidity in care we check blood pressure, lipids at diabetes checks so we are keeping on top of it. (HCP 12)*  *We have two separate hospital teams special for cardiac and diabetes and even when we go to the cardiac team on the ward they still don't know hardly anything about diabetes like we're having to teach them more. Whereas again myself, I don't know a lot about the cardiac, but I do think it would work that where if in future we could have monthly meetings where we could even share our knowledge, learn off each other, that would be really good. (HCP 10)*  *They don't even know what Hba 1C is and then they don't know where they stand. And you're right, they don't even ask you what are my blood sugar levels today to know (HCP 14)*  *Proactive, quite strict that if people have more than one thing, we will review every single thing at that same review. So if there's someone that's hypertensive, diabetic dementia, CHD, COPD they get a review of everything in the one appointment and you… Yeah, that doesn't happen in a lot of places. And we can tell that when the notes come over and there's only one diary dates like for diabetes and like, what about the seven other problems theyve got? (HCP 15)* |
| *Inhibition of Meaningful Interactions* | ***Fragmented Appointment Structure*** | *Yeah. I mean, I think the big barrier to all of this, and personalised care is time. (HCP 3)*  *Cardiovascular leader taken on in the last couple of years and haven't done as much as we would like with that just because of time as much as anything (HCP 3)*  *Encouraging to manage them and take responsibility and kind of to do a lot more than lifestyle side of things. But yeah a lot of it's just time really, but yeah. (HCP 3)*  *No, I mean obviously we can chat to them, but our time is limited here. (HCP 3)*  *The big problem with the counselling support is is time. (HCP 3)*  *In terms of specifics I probably don't go too heavily into that cause I just don't have the, you know, the time to do that. So yeah. (HCP 4)*  *Yeah. Yeah, time is is, you know, certainly one of the big things (HCP 4)*  *Erm it’s never enough time, there’s never enough (HCP 5)*  *Time, our appointment slots, and one of our nurses is leaving and obviously the QOF, flu season and covid season all added in and stresses like that all add in (HCP 6)*  *Yeah time, that’s the thing its just time, its having time with the right people erm you know first and foremost I don’t know what they do in 10 minutes I honestly don’t and then the time constraints on practice nursing or a nurse associate and there’s so many different things I am loosing track myself now, if its someone who knows what they’re talking about then that’s who the person needs to be sat in front of so erm and also given the opportunity to ask questions, but everything is a time constraint, but you know even if you know you had a drop in clinic you would have people drop in you know something they want to ask its….its giving the patient the permission and the opportunity (HCP 5)*  *I feel at present family doctors only have 10 minutes per appointment don’t they to see patients, 10 minutes allocated, and patients come with a magnitudinal amount of issues don’t they sometimes. You know you can see on Emis they’ve got erm gaggling of wrist, weight management, haemorrhoids ….*laughs* they’ve got a magnitudinal and you just thing wow. You’ve probably not had a good quality conversation about the management of anything in that 10-minute appointment….so where we come into play as health coaches, we allow up to 45 minute for appointment and that allows more often than not allows patients to sing like a bird…like wow so all the problems that have been bothering them which all of them are often problems for family doctors but they can’t always get the time they need, family doctors are struggling (HCP 1)*  *Yeah. No, I think I think a lot of a lot of times we are all hiding away behind the time limiting factor. Time is…..time is often an excuse for poor care (HCP 9)*  *The lack of capacity, for face to face appointments are massive at the minute. Near enough that’s possibly the most common theme that I can identify. (HCP 1)*  *I don't really have as a family doctor, I don't have a long time with my patients and we struggle for continuity because we've got a lot of trainees (HCP 13)*  *So having that approach, I think is probably not within the skill set or you know the abilities of the family doctor because we're dealing with more sort prescribing complexities and looking at other things maybe, but I'm definitely interested and I think if we're also enough the same hymn sheet, you know it's one thing (HCP 12)*  *I mean I think because of the time limits you have 10 to 15 minutes if you are lucky with a patient….lifestyle doesn’t always get discussed with the patient and then we can put them in touch with lifestyle diabetes education but even that sometimes isn’t on or gets cancelled or these people cannot make the date because they are in work and we don’t have the time so we do refer to the nurses who do their 3 monthly checks and the patient has the reviews (HCP 12)*  *I think it’s a package really…you need to know their background and you need to know everything and I think in a 10 minute appointment slot you cannot do this and erm we try to work around and what the number is telling us and erm it’s a bit difficult to do that (HCP 12)*  *Time constraints are one, you know as discussed we don’t have time to discuss with patients what they all need to know and if they are sitting there trying to take this all in are they actually listening to us or just trying to process a diagnosis so with that things become lost in translation and erm you know they then also may feel they cannot ask any questions as the time limitation and you know answering questions helps us as professionals figure out the needs of the patient and also gives them the ability to understand more but it is time it really is the most major barrier. (HCP 12)*  *Erm I don’t know, I mean I suppose that is another barrier, time, time with healthcare for patients (HCP 11)*  *Well I mean it’s something that needs to be done, you know making sure there lipids are checked, there bloods are checked, all those things are important and I think having these templates help that but your question is if it gets in the way of what’s important for patients well maybe I don’t know…..(HCP 11)*  *Yeah. So. So yeah, there's perhaps that is a barrier (templates) and it probably is a barrier (templates) and but I kind of just side step it (HCP 4)*  *Oh I cannot bare them, I cant stand them, (HCP 5)*  *But if I started with them I don’t think I would look at the patient once because I am too busy trying to look at what line I need to fill in.... and that’s the thing it prevent you using your initiative and talking to your patients and erm I don’t like them, so the patient might not even drink and you have to fill out that…..and there are comments on these templates that mean nothing, its just a tick box and you look at that line and you think what relevance is that line but it means nothing, I just think….. You have to fill out so for QOF for you do, I mean don’t get me wrong they can be very useful but erm they should I feel they should be use as a prompt and not as a erm you know a step by step this is what (HCP 5)*  *I wonder whether the move for these templates is because they’ll have erm erm not untrained or whatever but maybe support workers or healthcare assistance and they will be sitting asking these questions purely for someone to look over and decide whether you know whether there needs to be intervention, that’s where I worry where that’s going (HCP 5)*  *Not really, so if you are having a generic appointment with someone about their blood pressure and you automatically trigger BMI calculator which is great saves clicking height clicking weight you know it is really good in that sense, but at the same time, there’s no, its not very fluid, there’s not much room for complementing other conditions, it would be good if it could pull through to emis you know a patient profile per say and the templates could be identified as per issue they’ve got as per active problem on emis you know like it would be really good if like you could trigger someone whose got hypertension essential hypertension so the system knows they’ve got high blood pressure then type 1 diabetes, type 2 diabetes instantly a template could be used to run multiple things quick and you could scan over, that would be really good, it would be more personalised. Because right now you’re relying too much on human touch to go in and say run that template, run that template (HCP 1)*  *I am actually doing a presentation at the moment, to deliver a workshop to the other nurses and team about how to do the NHS health checks from my opinion I suppose but from a more personalised perspective which includes being quite flexible with the template on emis, so I’ll make some suggestions within that workshop about some of the core, what we should always include but yes there’s a lot of boxes with questions with ticks that have a lot to do with template that take time so my suggestion in that workshop is for them to be much more flexible within that workshop, do you need to go through a whole alcohol audit if they drink you know relatively little, for example, could you save that five minutes and talk about yeah a personalised approach instead (HCP 2)*  *I think it’s the user needs to be flexible and have confidence they don’t actually, there’s going to be no ill repercussions of not following a template by the letter, I think a template is helpful as it prompts your memory you know particularly if you aren’t doing something all the time, for example it is a good memory aid to make sure you ask key questions and gives confidence but also flexibility to be, to choose elements of it without being…..yeah (HCP 2)*  *But the fact that they're all in disease profiles means that you are still dealing with singular disease profiles, so there's no proof target for comorbidities, and also it's quite duplicating because it means that one patient will come in for three or four different disease and reviews, heart failure, diabetes, hypertension, COPD but if there was actually comorbidity clinics that were an hour and the outcome was you were going to get all four of those things done and the patient was going to learn that they're all interconnected and that lifestyle is the key to that. (HCP 13)*  *I don't think things have changed for a long time. I think we're still run diabetic appointments. We're still run COPD appointments. They're still at the same length of time and nurses have slightly different interests, and it is very important that, you know, we have a good nursing team but we've got one that does diabetes and another that does something else…they could be merged into one….but it takes a lot of time and training and experience from your workforce as well to be able to do it. (HCP 13)*  *Erm I mean no *laughs* that is the short answer you know there is template after template but they are for a singular disease I mean we get notifications to check blood pressures and things but I wouldn’t say collectively there is a template you know (HCP 12)*  *It would be actually a lovely idea, I think, to have more focus on comorbidity type clinics because I think the way that we run chronic disease is quite separate. I think the cross system that we use in community care looks at disease profiles in a very separate way. So when patients have their diabetic review, they go and see the nurse and they have their diabetic review and their diabetic review is geared towards diabetes. It’s geared towards foot checks and eye checks and then they go and have their hypertension review and their hypertension review is geared towards hypertension, getting the bloods, making sure the blood pressures and control but those two things are very closely related, but because patients are being dealt with them in different disease clinics, that doesn't help them make the connection between the two and how one can impact on the other (HCP 13)*    *Yeah. I mean, I think it all kind of forms part of it. And I think the templates, yes, they can be a bit of a tick box exercise. (HCP 3)*  *An example that's recently come out is in our clinical system there is a new diabetic sort of highlight you know the little alert box and it actually has nine points of care And for each patient. So it's useful to have that sort of reminder for the clinicians, it's like, oh, hang on, you're up to date with or you've had your vaccines or you've had your blood pressure's checked or your target your weight, you know, whatever the case may be. So it's useful and it's hard because there are so many conditions but but certainly that is a really useful tool that you can just add one glance look. Oh, hang on, you're up to date with this. You've done that you don't, you know, just keep people on track because healthcare professionals are busy and but tools like that will aid, you know (HCP 9)*  *you automatically trigger BMI calculator which is great saves clicking height clicking weight you know it is really good in that sense, but at the same time, there’s no, its not very fluid, there’s not much room for complementing other conditions, it would be good if it could pull through to emis you know a patient profile per say and the templates could be identified as per issue they’ve got as per active problem on emis you know like it would be really good if like you could trigger someone whose got hypertension essential hypertension so the system knows they’ve got high blood pressure then type 1 diabetes, type 2 diabetes instantly a template could be used to run multiple things quick and you could scan over, that would be really good, it would be more personalised. Because right now you’re relying too much on human touch to go in and say run that template, run that template (HCP 1)*  *I am actually doing a presentation at the moment, to deliver a workshop to the other nurses and team about how to do the NHS health checks from my opinion I suppose but from a more personalised perspective which includes being quite flexible with the template on emis, so I’ll make some suggestions within that workshop about some of the core, what we should always include but yes there’s a lot of boxes with questions with ticks that have a lot to do with template that take time so my suggestion in that workshop is for them to be much more flexible within that workshop, do you need to go through a whole alcohol audit if they drink you know relatively little, for example, could you save that five minutes and talk about yeah a personalised approach instead (HCP 2)*  *I think it’s the user needs to be flexible and have confidence they don’t actually, there’s going to be no ill repercussions of not following a template by the letter, I think a template is helpful as it prompts your memory you know particularly if you aren’t doing something all the time, for example it is a good memory aid to make sure you ask key questions and gives confidence but also flexibility to be, to choose elements of it without being…..yeah (HCP 2)*  *So I think when they first came in 15 to 20 years ago, it was very, you know, everything was based on hitting these targets. I think that's there's less burden of that, but there's still a burden. And, but the fact that they're all in disease profiles means that you are still dealing with disease profiles, so there's no proof target for comorbidities, giving lifestyle advice about not our or not our colleagues activity disease. We do obviously have smoking and alcohol, but you know that's not the QOF targets aren't allowed aligned to came up bitties, which I don't think is that helpful. And also it's quite duplicating because it means that one patient will come in for three or four different disease and reviews, heart failure, diabetes, hypertension, COPD. There was actually we had come morbidity clinics that were an hour and the outcome was you were going to get all four of those things done and the patient was going to learn that they're all interconnected and that lifestyle is the key to that. That would be better, wouldn't it? (HCP 13)*  *I don't think things have changed for a long time and how we think about going well bit easier. I think we're still run diabetic appointments. We're still run COPD appointments. They're still at the same length of time on nurses have slightly different interests, and it is very important that, you know, we have a good nursing team to do. A lot of these general reviews, and so our nurses, we've got one that does diabetes and they could be merged into one….but it takes a lot of time and training and experience from your workforce as well to be able to do it. (HCP 13)*  *Erm I mean no *laughs* that is the short answer you know there is template after template but they are for a singular disease I mean we get notifications to check blood pressures and things but I wouldn’t say collectively there is a template you know (HCP 12)*  *It would be actually a lovely idea, I think, to have more focus on comorbidity type clinics because I think the way that we run chronic disease is quite separate. I think the cross system that we use in community care looks at disease profiles in a very separate way. So when patients have their diabetic review, they go and see the nurse and they have their diabetic review and their diabetic review is geared towards diabetes. It’s geared towards foot checks and eye checks and then they go and have their hypertension review and their hypertension review is geared towards hypertension, getting the bloods, making sure the blood pressures and control but those two things are very closely related, but because patients are being dealt with them in different disease clinics, that doesn't help them make the connection between the two and how one can impact on the other (HCP 13)*    *Yeah. I mean, I think it all kind of forms part of it. And I think the target, yes, they can be a bit of a tick box exercise. (HCP 3)*  *An example that's recently come out is In our clinical system there is a new diabetic sort of highlight you know the little alert box and it actually has nine points of care And for each patient. So it's useful to have that sort of reminder for the clinicians, it's like, oh, hang on, you're up to date with or you've had your vaccines or you've had your blood pressure's checked or your target your weight, you know, whatever the case may be. So it's useful and it's hard because there are so many conditions but certainly that is a really useful tool that you can just add one glance look. Oh, hang on, you're up to date with this. You've done that you don't, you know, just keep people on track because healthcare professionals are busy and but tools like that will aid, you know (HCP 9)*  *The appointments are short, and everything is structured around ticking off biomedical boxes (HCP 16)* |
|  | ***Disjointed Collaboration*** | *Sitting down and having that conversation with people does take time and you want to then be able to support them and follow them up. And we are lucky in this practice in this network that we've got, we have got the health coaches, they are great, we refer to them and we've got the social prescribers. So if they're having social issues, we can refer them to the social prescribing side of things in themselves. (HCP 3)*  *If they are needing CBT, cognitive behavioural therapy, counselling through issues like that, then we can refer into talking therapies . The health coaches again will support with anxiety, sleep management, that sort of thing. You kind of lifestyle change again, social prescribing if there are issues that they need to look at from the you know just practically in terms of what support is out there for them. So there are things (HCP 3)*  *Yeah, yeah, I think these days I think probably would pass that over to the health coach and certainly sort of touch on the, you know, on the basics and broad strokes. (HCP 4)*  *Outside the PCN we do have *insert local charities*, they tend to have support groups they have charities, erm they receive funding from the community, I try to signpost patients to the community because there is research to suggest that the more you engage in community-based things the more support you have around you the better outcome for your health so yeah. (HCP 1)*  *So as health coaches again depends on practice, especially around lipid, you know this isn’t hba1c but does tie in quite similarly we are now able to educate patients on lipid management, how to manage you lipids naturally, and I know some practices are implementing this with hba1c naturally reduce your blood sugars. (HCP 1)*  *I suppose how you can look at it I suppose is if the nurse….whose doing say the health check…or the diabetes review….you know…if they identify within that appointment a clinical need for health coaching then that is an opportunity for me then to pick something up like that. A prime example of that could be….erm a patient is sent over because there recent hb1ac is poorly managed and they’re struggling with weight, they’re struggling with motivation, they’re struggling with diet, they basically want diet and lifestyle advice….and often as part of the task or referral it will say…poorly controlled type 2 diabetes (HCP 1)*  *I think we are one of the only PCNs across the *insert area in Northwest of England* that have access to mental health resources in that sense, in terms of the practitioners and things like that. If that’s not appropriate, then they can access things like therapy from there family doctor. We would then task the family doctor or send an urgent or routine appointment dependent on the emotional needs on the patients. The family doctor would then look to refer them on from them (HCP 1)*  *Were lucky we have mental health co-ordinator teams so we have one that works here in the practice but then two others across the PCN and I can refer to them if more specialist mental health is needed, so I can talk about low levels but when it becomes a little bit more complex then those additional roles can take on…….(HCP 2)*  *Yeah I mean I think it has improved over the last few years as we have all the additional roles and things, the wellbeing practitioner and health coaches, that’s been really helpful to have people to refer to because people need….you know now with these roles they are more keen to come in and see or have a telephone call with someone who can support and those appointments have been really great (HCP 6)*  *I don’t consider myself an expert in any of them (long term conditions), and I don’t feel I need to, it’s just about conversations and giving people the space to talk (HCP 2)*  *I would say no. I think we do another thing and we're all sort of supporting different parts of the elephant in a way and we don't have big MDT meetings about, lifestyle you know we don’t have an hour together about lifestyle. You know What are you seeing? And then what are you seeing? What are we seeing? What can we do and just, you know, throw ideas around and get some general consensus about the direction of what, what we want to do so everyone's working in their own individual silos. (HCP 13)*  *Look we’re all in the same boat paddling towards the same goal, of supporting the patient to achieve best outcomes and better quality of life right but I personally feel they (health coaches) need to be medically trained as they’re giving NHS guidelines on diet and that which is great but is it personalised to their condition or medications or you know if they are on statins do the additional roles know what this means...I am not sure (HCP 9)*  *This is why I had to go to the dietitian clinic to sit in because I don't know what's going on. What are the dietitians telling my patients? (HCP 13)*  *I generally didn't have a clue until I had to go and sit with him. I don't know what's going on and it's not helpful for the patients if they're going to a health coach who saying don’t go low carb and then the comes to see me and I am saying do .So it's just, you know that's not helpful for patients to be getting so many different mixed messages and there isn't an opportunity for us to come together. (HCP 13)*  *I would say no. I think we do another thing and we're all sort of supporting different parts of the elephant in a way and we don't have big MDT meetings about, lifestyle you know we don’t have an hour together about lifestyle.....everyone's working in their own individual silos....but that that is the NHS, isn't it? You know, everybody's doing something that's slightly different with no general consensus about what direction you want to move in (HCP 13)*  *I have heard mixed reports as some clinicians really think they (health coaches) are useful but then some think how can they help if they are non-clinical....do they understand diseases and what that means for a patient to live with this disease and how this effects them you know whether that be arthritis or Angina or Diabetes....do they understand the diseases (HCP 13)*  *The extended roles with all due respect they are a part of the multidisciplinary team but they are not clinicians and sometimes they order things and we don’t know why they have done that and then things have been discovered accidentally and then add that to the load and erm (HCP 12)*  *Erm I mean again its hard it depends on the experience of the person doing it so I mean pharmacists can be useful as they do medication reviews and they can take a lot of pressure of us but I mean paramedics can do home visits but we then have to supervise you know as family doctors as we have to help them out you constantly have to be there to supervise and we aren’t allocated time for this so it could be in the way but again PCNs its working fine and others not…..I think that’s the thing you will talk to some and they will be like yeah its great and other ones not and then even for some patients its working great and some it’s not…..in my experience family doctors will some rely on these additional roles too much you know there are the health and wellbeing coaches who people can go to but these aren’t there to support those with long term conditions they are general health and diet but if they don’t know what the patient is going through and don’t understand the medication you know the physical activity recommendation may not be suitable so then they may push themselves to much become more ill you know breathless and then come back and want family doctor appointment (HCP 12)*  *Erm so yeah in all honesty it depends on practice to practice. I know some larger surgeries have good pathways in to us....I think I could potentially identify that to better my role.... a better referral pathway.....but as I say its clinician dependant, some are very for it and say wow yeah we can help with diet and we can talk about glucose and others are like that’s not a health coach role don’t get involved in that. (HCP 1)*  *Poor communication between…… various teams can be a little bit of a barrier it depends on……. You know, we could probably all communicate a little bit better (HCP 4)*  *I think there's that too many things going on that we lose track of the importance and I think the communication in community care is very poor and it's like we could be doing something really good in our area......We'll try and share it out, but it falls on deaf ears. All lost in translation and the Chinese whispers fall down the line (HCP 7)*  *So I think the communication is key and integration with the ICB and integrated care boards. Now we have to manage diabetes and all other populations with their different comorbidities but it's bringing them all together for, you know, the for the patient (HCP 7)*  *So it's patients got this illness, this diagnosis and this diagnosis, but we all need to work together to get the best because although you can copy everything for the patients as from a cardiovascular side, but then they'll go and see the diabetes and they will say oh no, don't focus on that you need to get this right. (HCP 7)*  *So the diabetes nurses will go in and they'll change their plan while they're in hospital with a patient and then they'll do a clear management plan for when they go home and then liaise with the family doctor and the diabetes team but I think sometimes that translation could be also lost …..so when the diabetic nurse within the community services comes to do the review they’re not picking up on the management plan that’s already in place so god knows what information they’re telling them (HCP 7)*  *And so it's really disjointed at hand, but if you get the communication clear and you and the patient is on board with you, they'll like have a really good management plan because I don't think we realise that the seriousness of it, especially when you got a cardiovascular condition and your diabetes is not controlled (HCP 7)*    *We do the basics we do smart and Diabetes UK and obviously if they need advice or support or disabilities or they have arthritis or poor mobility they can be referred to a health coach for more support. We use Diabetes UK booklets off the Diabetes UK website, we signpost them on to that. We do basic dietary advice, were not dietitians but you know we know the basics and we go through that with them. (HCP 6)*  *Yeah yeah they can go on the smart courses or can go see a dietitian. The smart courses aren’t great the waiting list is……yeah at *insert hospital in Northwest of England* yeah….it’s not the best service out there but it’s there but I think it needs to be and we’ve had this for years and years and they go on courses that it’s not the best time it’s in the day times sometimes there’s one of the evening and unless its full they cancel it (HCP 6)*  *But I think I think they're better with the kind of like advanced clinical practitioners, you know, and all the physical, what they called Physician Associates and all those kinds of additional roles that are a bit slower to come into fruition in the hospital. I think they're being used a bit more for this kind of long-term conditions, which is great (HCP 8)*  *Well, Gee, the best place to look at people more holistically, cause in hospital based care we are very siloed….So we are kind of very fragmented where I think community services and the teams there and will set up to look after multimorbidity and long term conditions. But that comes with them having to know they have to have good knowledge about these conditions to. (HCP 8)*  *If you're gonna look somewhere, it's community care because they aren't doing the job. Basically, hospital based care is acute We are like a stop gap. We call it. but they're seeing them patients there under their radar community care are getting paid to look after them patients and some of them aren't getting the best carers others. So we do call it a bit like the postcode lottery as well with different family doctors and how good they're care is the down to the postcode and I'll know without even assessing that they're care is poor and it's usually right, yes. Yeah, basically, like almost deprived area in *insert area in Northwest of England* is *insert location* and we'll see you the care from there Compared to *insert area in Northwest of England* , that's not even 50 minutes away. It’s completely different. (HCP 9)*  *if you're gonna look somewhere, it's community care because they aren't doing the job….. they don't cover scenarios, so a lot of our patients, when they're unwell, diabetics, they bring themselves here but they don't need to be here at all, they just need the correct sick day rules of telling them what they need to do if the blood sugars are consistently high, they don't know what to do and all average blood sugars are like 47. I've seen family doctor documents saying as long as they are under 20 it's fine…..it's not fine because they're going to have a heart attack in five years (HCP 10)*    *A lot of our community as well or too scared to initiate insulin so they get referred to come to A&E to start insulin which more than any doctor can start themselves, but they won't so. They're just, I think they're out of touch with their training and they just will not do it there and but they won't do anything without a letter .I will change a patients insulin because ones out of stock and they won't automatically change it without the help of the pharmacist…they want a written letter for me to say. Can you please put this on a different brand of the exact same drug…..They won't do it. Yeah, it's very old school that they have to have a letter to make any change, even though they are a family doctor in a prescriber themselves, whereas I am and only a nurse. But I have to tell them what to do. So I would think they need some changes, mainly in community care. (HCP 10)*  *It's just it’s too clunky and we work too differently. I mean we work on electronic health record and they mainly work off paper record and then we liaise with each other via letters and it’s a giant system that crosses both to manage diabetes care... they have no conception of what we do....yeah it's a big segregation and the patients fall in the middle (HCP 11)*  *I think what we are seeing in the last few years but they are doing more guidelines and more training for us in community care and that’s not what we trained for I mean we are managing more complex things and we can’t train and keep updated all the time and you feel like your pulling yourself in all these directions and it gets to a point where you say I really can’t I need to refer and then you get this sometimes you send letters and you worry that it maybe something they reject or they say some other guidelines to follow but I think they have more access to investigations and do things quicker….I think in community care time is precious and we don’t have time or resources and erm especially sometimes they feel angry as to why we have referred someone but they don’t understand what community care goes through or what the patient looks like when we saw them and I think it has been in the culture for a long time (HCP 12)*  *With hospital based care as they are meant to see people who have lots of other problems but they don’t get seen…we send referrals but they don’t get seen and then any issues they have they just get worse and then they come to us for a desperate you know is there anything else you can do for us and that increases in demands for other reasons (HCP 12)*  *So I work with two different teams and one team is great at communication and working together and the other one is just completely disconnected and you don't know anything about the patient that’s coming in and then everybody's saying, but you don't know what the other person said and not all clinics have dietitians, which is crazy for type 2 diabetes and we have a huge lack of also psychology in the clinics and we have desperate need for that (HCP 14)*  *And so I think the MDTs are not complete to begin with. And I think that, yeah, communication, it depends… like it's you and your luck which team you get into basically (HCP 14)*  *Yeah, it is very poor. Like, yeah, there's barely any communication. To be fair. I mean, now we've had such an overload of patients that we're having to because of the lack of healthcare professionals in this area, we're having to discharge people quite a lot…its so sad (HCP 14)*  *Like I say, we have no idea what's going on within secondary care until we get that letter, which might take weeks for it to be gets to us…it goes to secretary's…they write it up. It then gets posted out (HCP 15)*  *Do they do their foot check? Do they. But if they don't document it so you then have to bring them in and they are like well, why do I have to come to you when I already go see the diabetic specialist (HCP 15)*  *But then patients don't come in because they don't want to come in because they don't want to see two different people. But you don't know if they've had everything done (HCP 15)*  *You know what I would love to do as a clinician as somebody who is trying to support people...what do you want? What do you need? And almost have a menu of options ideally in the form of human beings to kind of say that...here is what we can offer you...some of the answers are actually really simple. (HCP 16)*  *I’d love to have more joined-up pathways. At the moment it feels like you’ve got the diabetes team over here, the cardiac team over there, and they rarely talk to each other (HCP 16)* |
| *Gap Between Understanding and Supporting* | ***Recognition Without Response*** | *There's a lot of depression. There's a lot of frustration. You, particularly with patients, you've had these conditions for a long time and you know it's a little bit easier, perhaps with newer diagnosis cause you kind of coming into it fresh. But when they've already battled with their weight for the last 20 years or, you know, they life is so busy that they can't do all their job, doesn't allow them the time, you know, it's it's trying to battle with that (HCP 3)*  *There's a lot of anxiety, a lot of depression, a lot of frustration on their part. It's frustration on our part as well in terms of what we can achieve for them, what we can offer them within the constraints of you kind of guidelines and medication and things so (HCP 3)*  *I do feel for them, even in new patients, but certainly patients who've had it a long time. And I think the weight is the big thing for a lot of patients. They feel that they can't get their weight down. There might be yo-yoed and dieted or their lives and trying to get them to then review things and look at things afresh is difficult (HCP 3)*  *Yeah, definitely, managing anxiety cause I suppose with lots of conditions, what's the the thing that you worry about when you've had something like that? Well, is it gonna happen again? You know, that’s the thing that make people worry (HCP 4)*  *You know, if they're continually worrying about it then and it's very difficult for them to to live, isn't it? And also it uses a lot of the family doctor time……You know, trying to reassure them (HCP 4)*  *That’s the thing diabetes is huge, and you can always give them something else, and that’s what I try and do anyway because if they have a bad erm if they have a disappointing result they can so I can imagine its so easy to feel disheartened (HCP6)*  *Ok so erm well for some…..do you mean well for some people with diabetes its there BMI a factor so some people might feel they don’t like the way they look because there BMI is on the high side….is that what you mean (HCP 5)*  *Yeah yeah yeah absolutely and it goes back to me saying before because it’s so relentless and they can so easily become disheartened and if they think they’ve been really trying for three months now and then they come and they’re Hab1c isn’t exactly great so absolutely on a erm on the emotional side of things yeah and also it’s a label, people don’t want to be labelled, one thing is a pet hate for me is you’re diabetic.....i hate that, I would say you have diabetes, so I think erm I think body image and just the fact that if they’re going out for meals they’ve got to urgh do I need to explain to someone what I am doing or what I’ve got….if they’re on insulin you’ve got to take meds with me, it is huge absolutely huge so yeah its one condition, its not even it’s the fact every time they put something in their mouth its guilt because they feel bad all the time so they want to give themselves a treat and then some will feel lots of guilt and I shouldn’t of had that and there’s a lot (HCP 5)*  *Yeah 110% so a lot of the patients I speak to before it gets to a stage, and I hate to say this but often when you see them and you think clinically there a stroke waiting to happen or wow they’re a heart attack waiting to happen and its often when they are at that stage that’s when they are the least engaged mentally, it's almost like they take it and put it to the back of my mind approach (HCP 1)*  *And from what I can see there’s definitely low levels of mood, low levels of motivation, low levels of you know, often you do pick up patients that have real little next to no support and they have no support networks around them. It effects the way they deal with their health, the way they engage, what access to resources they’ve got, getting outside the house, being able to confide in people, a lot of people don’t have that access so yeah you know (HCP 1)*  *Low moods, lack of confidence, feeling disempowered, yeah erm yeah (HCP 2)*  *I think a lot of people have anxiety erm just with having a chronic disease and day to day life they’re taking medication that they maybe don’t understand and don’t think they should have to take and they have anxiety around the complications but I think there’s a lot of generalised anxiety...it’s on the increase so a lot of people that come and see us already are heading towards anxiety and depression whether its new or existing (HCP 6)*  *Yes, I know. I think it's very useful because sometimes it's an opening and see conversations as well. A lot of people, especially older generation, don't want to speak about any, you know, mental health or psychological things . And yeah, and I just say, you know, everything it all goes hand in health hand.*  *Some people are very anxious as it is, and they just feel all doom and gloom and they've had no support but a lot of people who are not taking it seriously. I will say like this is what's gonna happen. (HCP 9)*  *Definitely we would say anyone who didn't say that they had diabetes, burnout would be lying. So many people have diabetes burnout, especially when they're on more medication like the insulin, and they have to think about every single thing they eat. Checking the blood sugars, they are definitely feeling some stress from having the condition we're seeing it a lot more (HCP 8)*  *Definitely not. When there's being twice a year, six months, they don't even touch on the mental side or the emotional side of having the condition. It's just a quick MOT to check how they're getting on (HCP 9)*  *More than anything, some anxiety as well, with patients starting new to medication and what the family will think and being on insulin and letting themselves down, they feel like when they have to go on their own medication because you can't diet control, that they've let themselves down. (HCP 9)*  *Yeah, they feel like it's their fault that they've done it, that they have to take insulin. It's their fault because they couldn't manage it properly. Like I had a dentist that ended up taking insulin and saying he doesn't want to be anymore here anymore. He feels like insulin is ruined his life so he was very upset and it wasn't even his fault of going on insulin it was his kidney function of not having the best control and support at the start. His kidneys are affected as well, so there's no other tablet could help control other than insulin but he definitely felt the depression after it (HCP 10)*  *A lot of them fear diabetes because it is such a complex issue that they do really worry about it and any changes they make, they definitely want input from somebody else and they don't want to do any decision making on their own, yeah. (HCP 10)*  *Umm depression. Depression is huge. Diabetes depression is huge. Diabetes burnout is huge because it's something that you can't put down. So you have it. You know, unless you go into remission. And even then you're really focused on the fact that this could return. So it's something that you think about all the time and that's very, very difficult. (HCP 8)*  *Lots and which came first, the chicken or the egg is a very difficult one to sometimes pick apart (HCP 13)*  *So do people get depressed because they've got lots of comorbidities? Or do people that have depression end up with more comorbidities, I think there's evidence to suggest both ways. But from my point of view, yes, it's people will often give up. Think why bother? And their life becomes more restricted. They have to do less, so they do less because they're not supported very well to do more. And so yeah, I think has a big impact on people's emotions and wellbeing (HCP 8)*  *I mean they might come to us in a separate appointment for this but you know I normally don’t see that as in they don’t come to me and link that in with their diabetes but again in the diabetes assessment they ask about wellbeing so the diabetic nurse normally goes through this with them so yeah I am sure you know diabetes burn out and depression and sometimes depression would make their diabetes worse and then they are more likely to have complications and the list goes on (HCP 12)*  *And if someone's got diabetes and now they've got a cardiovascular, you know, they're off the wall with new medications and psychologically it can look like a domino effect (HCP 7)*  *many said that they don't feel well equipped to address a patient's emotional needs and therefore feel reluctant to broach the topic…So obviously that's a big one (HCP 14)*  *So we are as well in that respect as we are not used to dealing with mental health issues as much as we don't know at all that much (HCP 15)*  *It leaves me feeling like I can’t do my job fully. We talk a lot about lifestyle, behaviour change, but if someone is feeling hopeless or anxious, advice alone doesn’t work (HCP 15)* |
|  | ***Passing Responsibility*** | *I mean I think the social prescribers are quite well established in my work and erm we have health box which is a low level CVD that is available in the communities and NHS talking therapies but I mean the wait list is long so what they do during that wait you know is a different story but you know it’s there its better than nothing but I suppose that’s all I really know for diabetes and I mean for CVD the same really. The messages are quite mixed really though as it’s not exactly emotional support for your conditions… I am not quite sure where you would find that (HCP 12)*  *In our place, we have for our patients that stay under us. But no, there's not really. It's up to the we ask the family doctor to refer one, but they haven't even got a built up service there anyway, so it is very difficult. We try and touch on it when we see them and ask how they're doing, how we can make it easier, but I don't think there's much what there. (HCP 9)*  *No, I mean obviously we can chat to them, but our time is limited here. If they are needing CBT, cognitive behavioural therapy, counselling through issues like that, then we can refer into talking therapies (HCP 3)*  *Yeah I mean we don’t really get involved erm obviously we can do the basics we can give them some information sheets and that but we would refer them on to the family doctor or to one of the practitioners, it’s not my specialised area at all (HCP 6)*  *We probably discharge them back to family doctor land (HCP 8)*  *You know they have a few sessions and then the person will say ohh well your kind of…off you go…but for a lot of them then needing more long-term support (HCP 3)*  *If they are exhibiting those kind of symptoms, we do have a referral service, but they're waiting list is quite long, so I do try and signpost them to some kind of self-help groups and peer support and all those kind of things that we are in our power to do and also talk to them about these kind of issues that is normal and that other people feel that way and don't don't blame yourself and you know it's I try and open and have conversations like that (HCP 8)*  *We can refer them on to places but mental health support is poor and mental health provision is poor and it used to be much better but it’s got a lot worse (HCP 11)*  *I think there’s two things when it comes to mental health I think first there is mental health and then there is behaviour change and I think there different and I think erm there’s not enough out there to support behaviour change….so our physician associate *insert name* does come from a psychological background and she can be more in tune to that but yeah there could be more to support behaviour change (HCP 11)*  *But in heart failure they can link in with the trust, but once they've been discharged and are an outpatient, it's always harder to get that support as an outpatient. So we have improved access to Psychological Therapies Team, but the wait time is a 6 to 8 10 week wait (HCP 7)*  *But since covid, we've had a few pilots now which are rare and we're just waiting on the outcome data then… now we are doing it and we come to the end of the pilot and there's no funds and we're going to be back to square one again. (HCP 7)*  *We asked if sign posting now more to it, kind of, um mindfulness type treatments, you know kind of headspace, calm, different acts and patients are more aware of that side of things. Um, yeah, it's hard. And at the end of the day, they have to want to engage with those things (HCP 7)*  *Yeah, there are obviously psychological support services that can provide support but not specifically for those conditions so sometimes that's a challenge and then sometimes people have had really bad experiences you know they've had cardiac arrests…and then they've had major surgery and they've been in hospital for, you know, been in ICU….and then they come out and there's not really anything to support them psychologically with that (HCP 4)*  *The psychologist can only see people with insulin anxiety, we need someone who looks at depression linked to diabetes. (HCP 14)*  *But basically, we really need a psychologist to look at everything else, because depression is linked to diabetes and we're not really targeting the root cause (HCP 15)*  *So that's any lot of gaps because I think we have one psychologist for the whole team… So it's like there's this huge gap where we're not targeting the root cause, I think and it's just so sad. (HCP 14)*  *Even simple things, like being able to offer stress-management sessions, or connecting people with others in the same boat—could make a huge difference. Sometimes the answers are simple, but right now, the system makes them complicated (HCP 16)* |

**Supplementary file D: Author Evaluation Tool Using the Reflexive Thematic Analysis Reporting Guidelines**

| **Advice for approach to reporting** | **Author comment / justification** |
| --- | --- |
| **Background and rationale** | |
| Provide a robust context and rationale for the proposed research in the introduction | The introduction was structured to address the main issues, identify gaps in the current literature and emphasise the limited perspective of qualitative research in relation to the research problem. |
| Clearly articulate a research question – one that is methodologically coherent | Following the robust introduction, the aims were logically structured and aligned with the chosen methodology. |
| **“Owning your perspectives”** | |
| Include information on guiding theoretical assumptions and other (e.g. explanatory) theory informing the use of TA | This study was guided by a critical realist ontological stance, recognising that while an objective reality exists (e.g., healthcare systems), individuals experience and interpret through social and contextual lenses. A contextualist epistemology also informed the approach, viewing knowledge as co-constructed between researcher and participant. These assumptions supported the use of reflexive thematic analysis, enabling an interpretative exploration grounded in lived experience while acknowledging the influence of broader structural factors and the researcher’s role in meaning-making. |
| Report in a way that is consistent with stated theoretical assumptions throughout | To stay aligned with the study’s critical realist and contextualist assumptions, all researchers acknowledged their influence on the research process, including topic selection, interview dynamics and theme development. An interpretive approach was also taken, using tentative language and avoiding generalisations, to reflect experiences as context-specific rather than universal truths. Themes were also linked to broader systemic factors, ensuring that findings were grounded in both participants lived realities and the wider social and structural context. |
| Evidence methodological coherence / integrity in both the research and the report | The study followed Braun and Clarke’s (2021) approach to Reflexive Thematic Analysis (RTA) for both data generation and analysis, ensuring this qualitative work was structured yet flexible. The report was also structured to align with RTA and justification for the why, how and when this approach was applied was also provided in the report. Additionally, based on the newly developed RTARG, the researchers created an evaluative tool to confirm rigor, transparency and trustworthiness throughout the analysis and reporting process. |
| Show evidence of reflexive practice | The heading of ‘Research Team and Reflexivity’ in the methodology section of the report notes the importance of all authors recognising possible unconscious bias due to their professional identities. |
| Write in a methodologically coherent style | The justification of why a qualitative methodology was chosen is discussed in the methodology section of the report. The report also followed the guidance outlined in Braun and Clarke’s (2021) approach to RTA throughout data collection, analysis and writing. Following this guidance in an organised manner allowed the researchers to effectively answer the research questions, whilst acknowledging their role in reflexivity and how their personal interpretations could cause potential bias. The researchers also created an evaluative tool based on the RTARG to support with making sure this guidance was followed coherently whilst still preserving the integrity of the analysis. |
| **Participants / data items** | |
| Describe selection of participants / data items | Participants were recruited through a partner community-care organization using a combination of email invitations, flyers, and social media advertisements. The specify type of sampling method that was employed to select individuals who met the study’s inclusion criteria was also noted. |
| Provide a rational or explanation around data set or participant group size / composition | The subheading of ‘Participants and Sampling’ in the ‘Methodology’ section of the report outlines how the study followed by Braun and Clarke’s (2021) review on data saturation, which emphasised the importance of choosing quality and depth of the data, rather than overly prioritising data saturation. |
| Discuss characteristics of participants / data items | Supplementary table 1 detailed the participants job role, professional qualification, gender, years of experience, if they worked in a community or hospital-based setting, ethnicity and the socioeconomic status of the practising area. The authors felt no other characteristic was needed to describe the participants in the current study. |
| Detail ethical approval and ethical code / principles followed, participants informed consent etc | The subheading of ‘Participants and Sampling’ in the methodology section provided details of the NHS Health Research Authority ethical approval code, and under the heading of ‘Data Generation’ in the ‘Methodology’ section also details how participants had to complete written consent before they could participate. |
| **Dataset generation** | |
| Provide some rationale for method(s) for data generation/data item sources chosen. | Throughout the report the authors provided reason as to why a qualitative design was needed as the methodological approach. In particular the ‘Design’ heading in the ‘Methodology’ section states that RTA offers a flexible yet rigorous approach for examining patterns of meaning in experiential data. |
| Describe development and/ or characteristics of data generation tool(s) | Supplementary file A provides detail of the interview schedule is provided/ Further, detail of characteristics of data generation tools such as encrypted audio recorder was also detailed in the ‘Data Generation’ heading in the ‘Methodology’ section of the report’ |
| Include details such as modality and/ or setting of data generation, time frame, and other pertinent procedural information | Details on where data generation was conducted, and the timeframe are detailed under the ‘Data Generation’ heading in the ‘Methodology’ section of the report. Braun and Clarkes (2021) guidance was used to generate semi-structured interview questions and prompts. |
| Describe who conducted any interactive data generation (which author or research role), and how. | The ‘Data Analysis’ heading in the ‘Methodology’ section details how and when the authors interacted with the data generation. |
| Describe the size/scope of dataset and dataset items. | The sub heading of ‘Participants and Sampling’ in the ‘Methodology’ section of the report provided detail of the sample size and job role. In addition, three healthcare professionals joined the study during analysis as second phase members to add scope and credibility to the findings. |
| Describe, and if relevant explain, any preparation of data for analysis. | The heading of ‘Data Generation’ in the ‘Methodology’ section described how audio recordings from encounters with participants were transcribed to make raw data files before analysis took place. Raw data files were also created from the discussions with second phase members; these were also analysed at a later date. |
| **Data analysis** | |
| Provide some rationale for use of RTA, and, where relevant, for combining RTA with other approaches and procedures | The ‘Design’ subheading in the methodology section provided detail as to why RTA was decided as the technique for the study. The use of reflexivity was also justified throughout the report. |
| Discuss how the researcher(s) engaged with the analytic process | The ‘Data Analysis’ heading in the methodology section of the report outlines the step-by-step process in generating initial codes and developing themes by the first author. These were then discussed and explored as a research team before being chosen. |
| Where more than one person is involved, describe who analysed the data (author or research role). | Authors were referred to by their order of appearance (e.g., first author) to indicate who analysed the data and at what stage, depending on the listing in the references. All researchers’ roles were further detailed under the ‘Research Team and Reflexivity’ heading in the methodology section of the report. |
| Use language to describe the process and products of RTA that is coherent with the values and assumptions of RTA. | The first author dedicated time to understanding the principles of RTA by reviewing relevant literature and guidance. The second author has an extensive background in qualitative analysis, which helped ensure that both the process and outcomes were flexible, reflexive and consistent with RTA’s emphasis on researcher subjectivity and interpretation. Further, the ‘Research Team and Reflexivity’ heading also noted how authors made a conscious effort to engage in self-reflection throughout the analysis, particularly after engaging with the data, which is in alignment with RTA’s values of acknowledging the researcher’s role in shaping analysis. Triangulation, second phase participants and PPIE were also used to enhance credibility and minimise bias in the findings. Further, Supplementary File B outlines the analytic process used and provides an overview of coding and theme development. |
| **Reporting the data analysis** | |
| Provide an overview of themes or thematic structure | The ‘Findings’ section of the report has an opening section that describes the themes and supported subthemes. |
| Ensure theme conceptualisation is appropriate to RTA, and any divergences are justified and explained. | The themes were not predetermined or rigidly applied, rather they were developed through an interactive process of reflective engagement with the data. This process was explained in the ‘Data Analysis’ section of the methodology. Furthermore, the application of the COM-B model as an additional framework for interpreting the inductive findings did not influence the conceptualisation of themes, as its use occurred after the inductive phase of the reflexive thematic analysis (RTA). This approach was justified throughout the report, as the COM-B model was considered to provide a novel behavioral perspective on cardio-metabolic care.  Further, all authors collaboratively examined, explored and reached consensus on the final theme names. Following guidance on RTA, there was also a conscious effort amongst authors to minimise the use of topic summaries as theme headings and shift more focus towards creating themes that have a shared meaning and that convey a story within the data. |
| Name themes appropriately | All authors recognized that names of themes should be both descriptive and interpretative and they should provide meaning. All authors explored and examined possible theme names and after triangulation and some reflection names were agreed on collectively. |
| Report themes in sufficient depth and detail. | The narrative of the themes and subthemes was crafted in a way to convey the underlying meaning of the data and its significance, ensuring that the findings were interpretated in a way that emphasised their wider implications. Each subtheme was also supported by direct extracts which were chosen for their ability to effectively support the explanation. Further, Supplementary File C provides an extended set of anonymized quotations and analytical interpretations supporting each theme. |
| Use subtheme judiciously | Subthemes were thoughtfully selected and only chosen when all researchers agreed they were relevant to the main themes and contributed meaningfully to the overall analysis. |
| Ensure the analytic narrative explains the meaning and significance of the data. | All researchers felt it was important that the analytical narrative was designed to illustrate the meaning behind the data and explain its relevance, ensuring the findings highlight their broader implications. Thus, all researchers were part of deciding what these interpretations meant throughout the write up period.  The ‘Discussion’ section further strengthened the analytical narrative by providing a more detailed interpretation of the findings, explaining how the COM-B model offered a novel lens for understanding the data, and drawing comparisons with existing literature while highlighting implications for future research. |
| Provide an appropriate balance of analytic narrative and data extracts – both data extracts and analytic narrative matter. | Braun and Clarke (2021) suggest that there is no perfect formula for balancing analytical narrative and data extracts. However, it is crucial that the themes are presented in a way that captures depth, detail and complexity. Following these guidelines, the authors had several discussions and agreed that at least three direct extracts should be used per subtheme. Depending on the depth of the subtheme, this number could extend to five or six, but it ultimately depended on the analytical flow. All authors reviewed the analysis section before it was finalised to ensure that the chosen number of extracts accurately reflected and supported the analytical narrative. |
| Demonstrate coherence between analytic narrative and illustrative/evidentiary data extracts. | The analysis followed a structured approach by which an analytical narrative was always used to introduce and explain a theme and subtheme. A direct extract was then used to provide evidence, showing how the healthcare professionals perspectives aligned with the analytical interpretation. |
| Integrate existing research and theory into the analytic narrative. | The ‘Discussion’ section of the report drew on the relevant literature to support (and sometimes challenge) the themes and subthemes identified in the data. |
| **Quality, evaluation, and conclusions** | |
| Draw analytic conclusions across themes. | The ‘Discussion’ section of the report provided an analytical conclusion across all themes and subthemes, and suggested their significance compared to existing literature and what this potentially meant for future studies. |
| Discuss implications or directions for future research | Based on the analysis, the ‘Policy and Practice Implications’ section presented recommendations informed by the COM-B model, focusing on building capability, enhancing opportunity, and strengthening motivation and culture. Collectively, these strategies offer direction on the development of a more sustainable, person-centred care system that balances clinical effectiveness with staff wellbeing and interprofessional collaboration. |
| Use and report quality practices coherent with RTA. | We used the RTARG as an evaluation tool to critically appraise the present study for methodological congruence and reflexive openness. |
| Evaluate the research from a Big Q standpoint. | The study explored and interpreted healthcare professionals understanding and perceptions of diabetes and cardiovascular disease as multimorbid conditions. The analysis provided in depth insights whilst the report adhered to the principles and rigor of the chosen qualitative approach. The researchers felt the findings provided a meaningful contribution to literature on healthcare professionals perceptions on type 2 diabetes and cardiovascular disease as multimorbid conditions in care, whilst also recognising how their roles might have shaped this interpretation. |
| Include reflections on research process and practices, including researcher reflexivity. | The heading of ‘Research Team and Reflexivity’ in the methodology section of the report acknowledges prejudices, biases and assumptions that may occur due to the authors job roles and preexisting beliefs. Also, the subheading of ‘Strengths and Limitations’ in the ‘Discussion’ section highlighted the authors reflections on the research process and practices. |
